# A common gene expression signature in Huntington’s disease patient brain regions

**DOI:** 10.1186/s12920-014-0060-2

**Published:** 2014-10-30

**Authors:** Andreas Neueder, Gillian P Bates

**Affiliations:** Department of Medical and Molecular Genetics, King’s College London, London, SE1 9RT UK

**Keywords:** Neurodegenerative diseases, Huntington’s disease, Transcriptional dysregulation, Network analysis, Therapeutic targets

## Abstract

**Background:**

Gene expression data provide invaluable insights into disease mechanisms. In Huntington’s disease (HD), a neurodegenerative disease caused by a tri-nucleotide repeat expansion in the huntingtin gene, extensive transcriptional dysregulation has been reported. Conventional dysregulation analysis has shown that e.g. in the caudate nucleus of the *post mortem* HD brain the gene expression level of about a third of all genes was altered. Owing to this large number of dysregulated genes, the underlying relevance of expression changes is often lost in huge gene lists that are difficult to comprehend.

**Methods:**

To alleviate this problem, we employed weighted correlation network analysis to archival gene expression datasets of HD *post mortem* brain regions.

**Results:**

We were able to uncover previously unidentified transcription dysregulation in the HD cerebellum that contained a gene expression signature in common with the caudate nucleus and the BA4 region of the frontal cortex. Furthermore, we found that yet unassociated pathways, e.g. global mRNA processing, were dysregulated in HD. We provide evidence to show that, contrary to previous findings, mutant huntingtin is sufficient to induce a subset of stress response genes in the cerebellum and frontal cortex BA4 region. The comparison of HD with other neurodegenerative disorders showed that the immune system, in particular the complement system, is generally activated. We also demonstrate that HD mouse models mimic some aspects of the disease very well, while others, e.g. the activation of the immune system are inadequately reflected.

**Conclusion:**

Our analysis provides novel insights into the molecular pathogenesis in HD and identifies genes and pathways as potential therapeutic targets.

**Electronic supplementary material:**

The online version of this article (doi:10.1186/s12920-014-0060-2) contains supplementary material, which is available to authorized users.

## Background

Huntington’s disease (HD) belongs to the group of poly-glutamine (polyQ) repeat expansion diseases, which together comprise the most common form of inherited neurodegenerative disorders [1]. It can also be categorized as a proteinopathy, a disorder in which abnormally folded proteins cause disease by loss- and/or gain-of-function mechanisms. Many other neurodegenerative diseases also belong to this category. For example aggregating proteins include the amyloid-β peptide (Aβ) and tau (MAPT) in Alzheimer’s disease (AD) [2] and α-synuclein (SNCA) in Parkinson’s disease (PD) [3]. The major aggregating proteins in amyotrophic lateral sclerosis (ALS) are superoxide dismutase 1 (SOD1) [4], TDP-43 (TARDBP) [5] and FUS [6]. However, other diseases that are not associated with misfolded proteins can also result in major neurodegeneration. Amongst these are brain tumors, e.g. gangliogliomas (GG) [7], which arises from brain ganglion cells, and inflammatory diseases such as multiple sclerosis (MS) which can result in a massive loss of neurons [8]. Furthermore, there is evidence that even very heterogeneous mental illnesses, such as schizophrenia (SCHIZ) are at least partly associated with neurodegeneration [9].

Whilst many of the above diseases are characterized by mutations in protein coding regions, mutations can also exert deleterious effects through RNA molecules. A hexanucleotide repeat expansion in the uncharacterized gene *C9orf72* is the most common cause of familial and sporadic ALS, as well as frontotemporal lobar degeneration (FTLD) [10,11]. The repeat expansion is located in intron 1 of *C9orf72*, thereby making it an RNAopathy, i.e. a toxic gain-of-function of an RNA leading to disrupted protein and/or RNA homoeostasis [12]. RNA gain-of-functions also occur in other repeat expansion diseases such as myotonic dystrophy type 1 (DM1) and type 2 (DM2) [13]. In DM1 the *DMPK* gene harbors a large repeat expansion in the 3’ untranslated region [14–16]. In DM2 the repeat expansion is located in intron 1 of the *ZNF9* gene [17]. The splicing factor MBNL1 is recruited to the repeat expansion in both cases [18], which in turn leads to a disruption of general mRNA processing resulting in cytotoxicity. Intriguingly it was recently shown that in HD a short transcript of the *HTT* gene is produced by aberrant splicing, probably influenced by abnormal binding of the splicing factor SRSF6 to the CAG repeat expansion [19]. In addition to the alternative splicing of *HTT* itself, other aberrantly spliced transcripts can be found in HD mouse model tissue (Gipson TA and Housman DE, unpublished data).

Transcriptional dysregulation, or a global change in gene expression is a hallmark of many neurodegenerative diseases, including HD, AD, PD and ALS [20]. For HD there is some evidence in patients [21–23] and mouse models [24,25] that these changes occur in the prodromal stage, which could make them useful to define disease progression on a molecular level, or even as potential biomarkers for therapeutics. Intriguingly, mutant huntingtin (HTT) itself was found to exert abnormal DNA binding activities [26]. The authors proposed that mutant HTT binding could alter DNA structure or sterically block access by other transcription factors and therefore be the initial cause of HD transcriptional dysregulation. The biggest study to date of human samples analyzed 44 HD patient and 36 control brains [27]. They found extensive changes in the caudate nucleus (CN) and BA4 region (motor functions) of the frontal cortex (FC-BA4). Almost no changes were found for the BA9 region (association, cognitive functions) of the frontal cortex (FC-BA9), or the cerebellum (CB). In a follow up study, the same group showed that the changes seen in HD patients were largely comparable to changes seen in HD mouse models [28].

However, standard evaluations of large, multi-dimensional gene expression datasets need to apply very strict statistical thresholds to correct for family wise errors stemming from the very high number of multiple comparisons. In doing so, small and/or maybe more heterogeneous expression changes may not be detected. Yet these small changes could contribute to an overall functional deficit, if they for example are all part of a certain molecular pathway. Alternatively, they may represent large changes in a subpopulation of cells. One solution to this problem is to analyze the data with correlation networks, which provide a more systemic view, instead of a per gene assertion. Weighted gene correlation network analysis (WGCNA) is a package of R functions, which allows one to construct such networks [29]. In these networks, groups of genes, which highly correlate in their expression, are clustered into modules. Next, these modules can be correlated to external traits, for example disease stage, age, sex, etc. Because only a small number, usually in the range of 10 to 30 modules per network, are identified, multiple comparisons are greatly alleviated. Another huge advantage is that one can detect “hub genes”, i.e. genes that are the highest connected genes in a particular module and are therefore most likely the biological key drivers. These hub genes also present bona fide therapeutic targets and/or biomarkers. WGCNA was successfully used to analyze many large datasets, noteworthy in the identification and cross-species comparison of brain region networks [30,31] and in the analysis of gene expression changes in ALS [32] and AD [33,34].

Here, we used WGCNA to study the transcriptional dysregulation in HD. To this end we constructed and compared networks for 4 different regions from patient brains and analyzed their preservation in gene expression datasets of other diseases, as well as in mouse models of HD. We constructed consensus networks of HD and other diseases to highlight common changes. These approaches allowed us to identify a common signature of transcriptional dysregulation in all three brain regions and to pinpoint potential future therapeutic targets.

## Results

### Weighted correlation network construction using WGCNA in the HD dataset

For a more detailed explanation of the WGCNA package, the interested reader is referred to the original publication [29] or the WGCNA homepage: http://labs.genetics.ucla.edu/horvath/CoexpressionNetwork. As outlined in the materials and methods section, we constructed weighted, signed correlation networks from the pre-processed datasets. Next, we identified modules that correlated to disease stage (in the following referred to as correlation with HD). To this end, we converted the neuropathological stage assignment of the samples, as listed in the original publication [27], to a numerical scale with controls as 1, HD grade 0 as 2, HD grade 1 as 3 and so forth. ‘Module eigengenes’, which represent a summary for all genes within a module were computed and subsequently correlated with HD. Negative, or positive correlation indicates that the expression of the genes in a module is lower, or higher, respectively, in patient compared to control samples. From here on we focused only on significantly correlated modules (Benjamini-Hochberg corrected *P*-values <0.05). Using ‘eigengene based connectivity’ (kME) as a measure of gene co-expression strength in a particular module (weighted from 0 to 1), we identified the genes with the highest connectivity in a module (hub genes). We also analyzed the preservation of the significantly correlated modules in a particular network in the datasets of the other brain regions. Preservation can be seen as the similarity of co-expression between genes in a module, but also connectivity patterns of individual modules for the two data sets. Preservation was calculated using permutations of the preservation statistics and is represented by a Z-summary value. High preservation, or high Z-summary values indicate that modules are densely connected, distinct and reproducible. Z-summary values tend to be higher for larger modules, i.e. small modules are very often found to be only weakly preserved.

**Figure 1:**
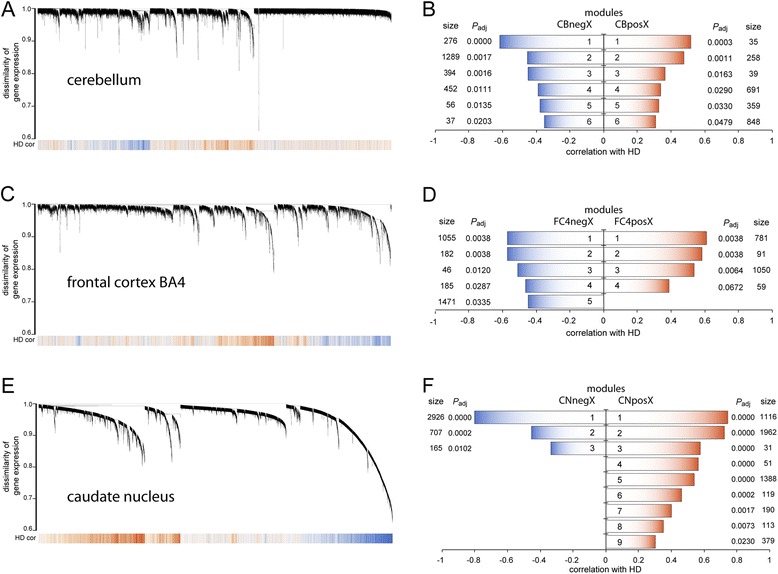
**WGCNA analysis of the HD dataset identifies highly correlated modules for each brain region. (A and B)** WGCNA analysis of the cerebellum dataset. **(C and D)** WGCNA analysis of the frontal cortex BA4 region dataset. **(E and F)** WGCNA analysis of the caudate nucleus dataset. **(A,** **C and** **E)** Hierarchical cluster tree of the average linkage in the dissimilarity topological overlap matrix. Each vertical line correlates to a gene. The height is a measure for the dissimilarity based on the topological overlap. The band under the dendrograms indicates the correlation with HD (HD cor) based on the gene significance for each gene. Red is positively correlated with HD stage, blue is negatively correlated. **(B,** **D** **and F)** Visualization of modules that are highly correlated with HD. Size is the number of genes for each module. *P*_adj_ gives the Benjamini Hochberg corrected significance value of correlation with HD for each module. Modules are labeled according to the network (CB - cerebellum, FC4 - frontal cortex BA4 region and CN - caudate nucleus), the sign of the correlation (neg - negatively correlated and pos - positively correlated) and ordered by *P*_adj_ with 1 being the most significantly correlated module, followed by 2, etc.

**Figure 2:**
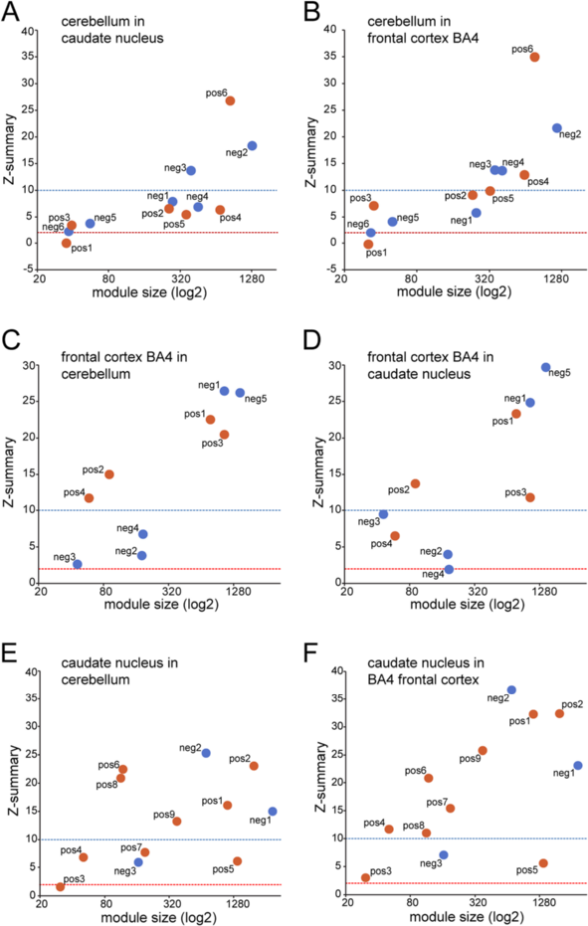
**Preservation analysis shows only few tissue specific modules.** The Z-summary is a measure for module preservation. Values less than 2 (red lines) indicate no preservation, between 2 and 10 (blue lines) module structures are preserved and above 10 the module structure is highly preserved. Preservation analysis of cerebellum modules in the caudate nucleus **(A)** and frontal cortex BA4 region dataset **(B)**. Preservation analysis of frontal cortex BA4 region modules in the cerebellum **(C)** and caudate nucleus dataset **(D)**. Preservation analysis of caudate nucleus modules in the cerebellum **(E)** and frontal cortex BA4 region dataset **(F)**.

**Figure 3:**
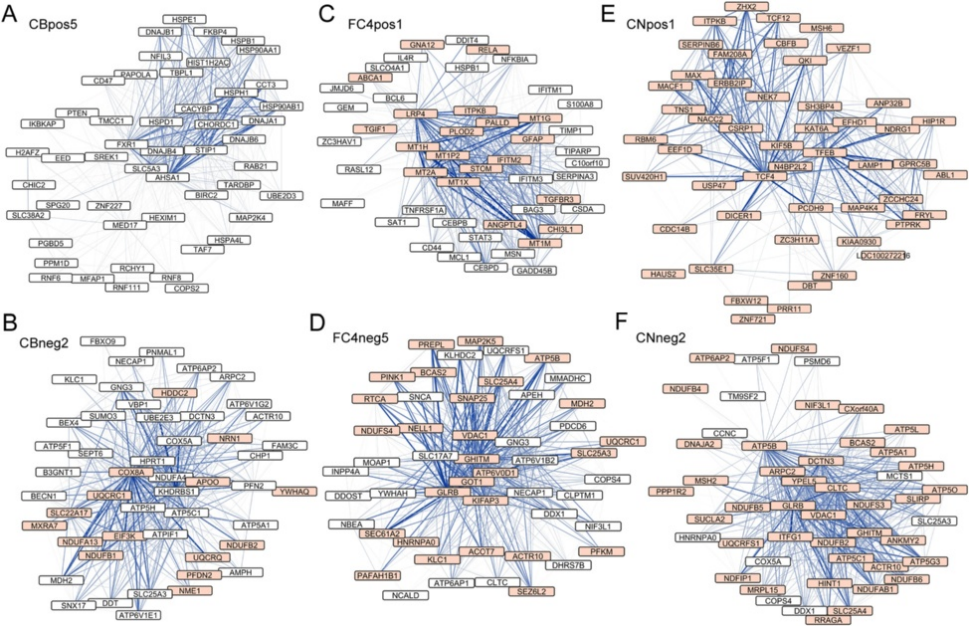
**Visualization of hub genes in network modules. (A -** **F)** The 50 most connected genes (nodes) and the 500 strongest gene-gene interactions (edges) in each module are shown. The width and the color saturation of the lines (edges) correspond to the weight of the interactions. The orange highlighted nodes correspond to genes that were also statistically significantly dysregulated [27]. Hub genes have a high gene significance value, as well as high eigengene based connectivity (kME). The correlation of both is shown in Additional file 1. **(A)** and **(B)** show hub genes from two cerebellum modules. **(C)** and **(D)** show hub genes from two frontal cortex BA4 region modules. **(E)** and **(F)** show hub genes from two caudate nucleus modules. The remaining hub genes for the other modules of the three networks are shown in Additional files 2, 3 and 4.

In the cerebellum dataset, 2504 genes (about 20.0% of all genes in the dataset) were assigned in negatively correlated and 2230 genes (about 17.8% of all genes in the dataset) in positively correlated modules (*P*_adj_ < 0.05) (Figure 1A and B). This is in marked contrast to what was found by Hodges and colleagues, who identified only 340 statistically significantly dysregulated probe sets, corresponding to 290 genes (HG-U133A chip; *P* <0.001). For gene ontology (GO) enrichment and regulatory factor prediction of the modules in the cerebellum network see Table 1. The eigengene based connectivity (kME) for all identified modules showed a significant linear positive or negative relationship with the gene significance for HD. Some genes exhibited high kME values and also high gene significance values indicating these genes are potential hub genes (Additional file 1A). The cerebellum modules CBpos5 and CBneg2 were highly preserved in both the frontal cortex and caudate nucleus. Modules CBpos4, CBpos5 and CBneg4 were better preserved in the frontal cortex, than in the caudate nucleus dataset (Figure 2A and B). Figure 3A and B illustrate the connectivity between the top 50 hub genes for the CBpos5 and CBneg2 modules (see also Additional file 2). 33 (66%) of the top 50 hub genes of the CBneg1 module were also statistically significantly dysregulated, as determined by Hodges and colleagues. Additionally, only modules CBneg2 (15/50 = 30%) and CBneg3 (14/50 = 28%) showed considerable overlap of module hub genes and dysregulated genes. In total, 46 (15.9%) of the statistically significantly dysregulated genes were not sorted into modules that were correlated with HD. The CBneg1 module is highly negatively correlated with HD (Figure 1B), but GO analysis showed no significant enrichment (Table 1). However, in depth analysis of the molecular function of the hub genes in this module revealed several genes involved in synaptic function. For example *CBLN1* is a cerebellum specific precursor of cerebellin, which is enriched in the post-synapses of Purkinje cells. Further neuronal related hub genes were *SLC17A7, SCN1B* and *PDE10A*. The other modules that were negatively correlated with HD in cerebellum are highly enriched for mitochondrial and proteasomal genes, indicating an attenuated function of these two processes (Figure 3A, Additional file 2 and Table 1). The positively correlated cerebellum modules are enriched for transcriptional regulation, chromatin binding/remodeling/modification, RNA binding/processing and metallothioneins (Table 1). Analysis of the CBpos5 module revealed a very high enrichment in protein folding/chaperone genes, as well as chromatin assembly and mRNA processing genes (Table 1). Intriguingly, 14 of the top 50 hub genes of the CBpos5 module are involved in protein folding, all of which share very high connectivity (Figure 3A). Notably, there was no indication that genes involved in inflammation or the immune response are correlated with HD in the cerebellum.

**Table 1:** **Gene ontology enrichment for the cerebellum network**

**Module**	**cor**	**GO-term (DAVID)**	**Potential regulators**
CBpos1	up	metal binding/zinc-finger (0.84, 0.89)	MYOD (0.0314)^2^, ZIC2 (0.0314)^2^, E2F1/E2F4 together with DP1/DP2, or RB (0.0314)^2^, NFY (0.0314)^2^
CBpos2	up	DNA binding/zinc-finger (3.97, 0.003)	miR124 (0.054)^1^
		chromatin binding/remodeling (1.82, 0.096)	
CBpos3	up	ubiquitin protein ligase binding (1.1, 0.49)	
CBpos4	up	metal binding/zinc-finger (6.98, 0.000)	
		chromatin modification (4.8, 0.004)	
		RNA binding/processing (3.41, 0.03)	
CBpos5	up	protein folding/chaperones (6.77, 0.000)	**HSF1** (0.09)^1^
		chromatin assembly (3.19, 0.003)	**HSF1** (0.000)^2^, NRF1 (0.001)^2^, USF1 (0.001)^2^, E4BP4 (0.024)^2^
		mRNA processing (2.95, 0.034)	
CBpos6	up	metallothionein (4.24, 0.003)	miR124 (0.027)^1^, let7 (0.027)^1^, SOX2 (0.027)^1^, MYOG (0.027)^1^, HSF1 (0.044)^1^
CBneg1	down	synapse (2.14, 0.33)	
CBneg2	down	mitochondrion (10.44, 0.000)	NRF1 (0.108)^1^
		proteasome (3.13, 0.000)	E4F1 (0.000)^2^, PAX3 (0.006)^2^, ATF (0.007)^2^, ELK1 (0.012)^2^
CBneg3	down	mitochondrion (2.77, 0.007)	SF1 (0.000)^2^, ERR1 (0.001)^2^, PAX4 (0.001)^2^, TCF3 (0.002)^2^, ZBTB14 (0.046)^2^
CBneg4	down	mitochondrion (2.85, 0.005)	
CBneg5	down	endoplasmic reticulum (1.02, 0.95)	
CBneg6	down	cytoplasmic vesicle (0.88, 0.99)	
HTT	down	mitochondrion (5.3, 0.000)	

Gene ontology (GO) enrichment for the HD cerebellum network. Genes in the identified modules were analyzed using DAVID. The sign of the correlation (cor) with HD and the over-represented GO-terms are shown. The first number in brackets after the GO-term is the respective fold enrichment, the second number the adjusted *P*-value, as determined by DAVID. All significantly enriched (adjusted *P* <0.05) GO-terms are shown. In cases where no significantly enriched GO-term was identified, the GO-term with the highest fold enrichment is shown. Potential regulators of a module were identified using ^1^GO-Elite, or ^2^WebGestalt. Adjusted *P*-values are given in brackets after the name. Regulators that were identified by both tools are highlighted in bold. HTT is part of the CBneg2 module in the cerebellum network. The GO-term enrichment for 100 genes with the highest correlation with HTT is shown.

**Figure 4:**
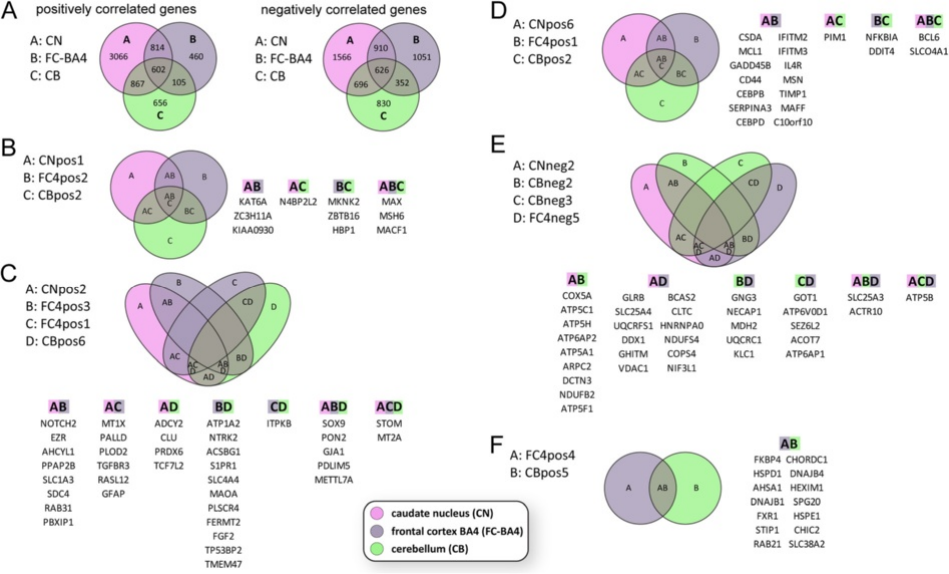
**Network comparisons between different tissues in HD reveal a high number of similarly correlated genes, as well as common hub genes in all three brain regions.** Venn diagrams show the overlap of networks **(A)** or hub genes **(B to** **F)** in the respective modules. Only modules with an overlap of more than 5 hub genes (10%) with modules from other tissues are shown. **(A)** Venn diagrams highlight the overlap of positively or negatively correlated genes in the networks of the three tissues. All positively, or negatively correlated genes of the significantly correlated modules (Figure 1) for each network were combined and compared to their respective assignment in the other networks. The intersections show the number of genes that were assigned to modules with the same sign of correlation. Caudate nucleus modules CNpos1 **(B)**, CNpos2 **(C)** and CNpos6 **(D)** are positively correlated with HD and have common hub genes with cerebellum and frontal cortex BA4 modules. Caudate nucleus module CNneg2 **(E)** is negatively correlated with HD and also shares common hub genes with cerebellum and frontal cortex BA4 modules. The positively correlated frontal cortex BA4 region module FC4pos4 **(F)** overlaps with cerebellum, but not caudate nucleus modules.

We could not identify any significantly correlated modules in the frontal cortex BA9 region dataset, the same result as obtained by Hodges and co-workers (data not shown). By contrast, we found significant changes in the BA4 region of the frontal cortex (Figure 1C and D). Here, 2939 (23.5%) genes were assigned to negatively and 1981 (15.8%) genes to positively correlated modules (*P*_adj_ < 0.05) (Figure 1D). For gene ontology (GO) enrichment and regulatory factor prediction of the modules in the frontal cortex BA4 region network see Table 2. As for the cerebellum network, the modules showed significant linear relationships between eigengene connectivity and gene significance for HD (Additional file 1B). Preservation analysis indicated that most frontal cortex BA4 region modules were equally well preserved in the caudate nucleus, or cerebellum dataset, respectively (Figure 2C and D). However, the FC4neg3 module was slightly less preserved in cerebellum, compared to caudate nucleus; modules FC4pos3, FC4pos4 and FC4neg4 were slightly better preserved in cerebellum (Figure 2C and D). Module FC4pos4, although it was just short of being significantly correlated with HD (*P*_adj_ =0.064) (Figure 1D), is, like the CBpos5 module in the cerebellum network, highly enriched for protein folding/chaperone genes (Tables 1 and 2, Additional file 3C and Figure 4F). The GO enrichment analysis of the other positively correlated modules more or less mirrored the cerebellum network with the exception of a high enrichment for inflammatory response and NFκB/IκB genes in the FC4pos1 module (Table 2). This finding was further supported by the fact that several transcription factors, which regulate immune response/inflammatory pathways, were identified as hub genes (Figure 3C). Amongst these were *CEBPB*, *CEBPD*, *BCL6*, *MT1G*, *NFKBIA*, *IFITM1*, *IFITM2*, *IFITM3*, *S100A8*, *IL4R* and *TNFRSF1A*. The FC4pos1 module was also enriched for genes implicated in angiogenesis, e.g. *SAT1*, *ANGPTL4* and *JMJD6* (Table 2 and Figure 3C). Notably, as for cerebellum, none of the negatively correlated modules was significantly enriched for synaptic/neuronal genes (Table 2). However, hub gene analysis of the FC4neg1 module (GO enrichment for synapse 1.86, *P*_adj_ = 0.063) showed several genes involved in synaptic/neuronal function (*GABRG2*, *SCN2B*, *RASGRF1*, *KCNJ9*, *GRIN2A*, *NRXN1*, *GPR176*) (Additional file 3D). Again, as in cerebellum, mitochondrial and proteasomal genes were negatively correlated with HD. Furthermore, genes implicated in protein transport, glycolysis and another set of protein folding/chaperone genes were assigned to negatively correlated modules (Table 2). The overlap of hub genes and statistically significantly dysregulated genes as determined by Hodges and colleagues was as follows: FC4pos1 20 of 49 = 40.8%; FC4pos2 3 of 50 = 6.0%; FC4pos3 38 of 44 = 86.4%; FC4pos4 1 of 50 = 2.0%; FC4neg1 28 of 50 = 56.0%; FC4neg2 10 of 50 = 20%; FC4neg3 10 of 45 = 22.2%; FC4neg4 3 of 50 = 6.0%; FC4neg5 28 of 50 = 56.0%. In total, 217 (28.9% of 750 genes) of the significantly dysregulated genes in the frontal cortex BA4 region were not sorted into modules that were correlated with HD.

**Table 2:** **Gene ontology enrichment for the frontal cortex (BA4 region) network**

**Module**	**cor**	**GO-term (DAVID)**	**Potential regulators**
FC4pos1	up	inflammatory response (6.64, 0.000)	MEF2 (0.041)^1^, NFkB (0.041)^1^, miR34 (0.050)^1^, let7 (0.041)^1^
		metallothionein (4.02, 0.04)	STAT3 (0.005)^2^, STAT5B (0.005)^2^, JUN (0.040)^2^
		regulation of transcription (3.58, 0.018)	
		regulation of apoptosis (3.25, 0.02)	
		vasculature development (2.7, 0.021)	
		cation homeostasis (2.68, 0.014)	
		IκB/NFκB (2.65, 0.02)	
FC4pos2	up	RNA binding/splicing (2.36, 0.037)	E2F (0.006)^2^, TLX2 (0.006)^2^, XBP1 (0.008)^2^, YY1 (0.011)^2^, HSF1 (0.021)^2^, LEF1 (0.021)^2^, MYC (0.021)^2^, SOX5 (0.025)^2^, AR (0.031)^2^
FC4pos3	up	amino acid catabolic process (5.52, 0.005)	miR155 (0.018)^1^
		fatty acid metabolism (3.25, 0.011)	MEF2 (0.038)^2^
FC4pos4	up	protein folding/chaperones (3.75, 0.001)	miR1 (0.009)^1^, **HSF1** (0.009)^1^
			**HSF1** (0.000)^2^, NFIL3 (0.033)^2^, STAT1 (0.037)^2^, SF1 (0.037)^2^, NFY (0.039)^2^
FC4neg1	down	protein transport (2.07, 0.03)	EVI1 (0.011)^2^, E4F1 (0.045)^2^, XBP1 (0.045)^2^, ATF2 (0.045)^2^
FC4neg2	down	membrane proteins (1.49, 0.2)	
FC4neg3	down	fibronectin (1.6, 0.84)	
FC4neg4	down	zinc-finger (1.74, 0.84)	
		mitochondrion (6.01, 0.000)	
		proteasome/ubiquitin system (5.01, 0.000)	**NRF1** (0.036)^1^
FC4neg5	down	glycolysis (3.5, 0.001)	ELK1 (0.000)^2^, SP1 (0.000)^2^, SF1 (0.001)^2^, E4F1 (0.001)^2^, TCF11 (0.005)^2^, ATF (0.007)^2^, JUN (0.007)^2^, **NRF1** (0.007)^2^, CREB (0.028)^2^
		protein folding/chaperones (3.04, 0.015)	
		protein transport (3.0, 0.002)	
HTT	n.a.	cytoskeleton (1.43, 0.18)	

Gene ontology (GO) enrichment for the frontal cortex BA4 region network. Genes in the identified modules were analyzed using DAVID. The sign of the correlation (cor) with HD and the over-represented GO-terms are shown. The first number in brackets after the GO-term is the respective fold enrichment, the second number the adjusted *P*-value, as determined by DAVID. All significantly enriched (adjusted *P* <0.05) GO-terms are shown. In cases where no significantly enriched GO-term was identified, the GO-term with the highest fold enrichment is shown. Potential regulators of a module were identified using ^1^GO-Elite, or ^2^WebGestalt. Adjusted *P*-values are given in brackets after the name. Regulators that were identified by both tools are highlighted in bold. HTT is part of a module, which is not correlated with HD in the frontal cortex BA4 region network. The GO-term enrichment for 100 genes with the highest correlation with HTT is shown. The GO-term enrichment for the frontal cortex network with BA4 and BA9 regions combined is shown in Additional file 11.

As in the original publication, we observed the largest changes in the caudate nucleus (compare Figure 1A, C and E), with 3798 (30.4%) genes assigned to negatively and 5349 (42.8%) genes assigned to positively correlated modules (*P*_adj_ < 0.05) (Figure 1F). While the correlation of modules in the cerebellum and frontal cortex BA4 networks was largely comparable, the correlation of the caudate nucleus modules was higher, highlighting the prominent pathology in the striatum of HD patients (compare Figure 1B, D and F). For gene ontology (GO) enrichment and regulatory factor prediction of the modules in the caudate nucleus network see Table 3. We again observed a very strong linear relationship between the eigengene based connectivity and the gene significance for HD, indicating approximate scale free topology of the network and existence of hub genes (Additional file 1C). Overall, preservation of caudate nucleus modules was better in the frontal cortex (BA4) than in cerebellum (Figure 2E and F). Again, this finding most probably reflects the degree of pathology in the different tissues. The CNpos6 module was the only module that was equally well preserved in both cerebellum and frontal cortex BA4. This module is highly enriched for inflammatory response genes (Table 3). The CNpos5 module seems to be caudate nucleus specific, as it was only weakly preserved in both, cerebellum and frontal cortex BA4 despite being a rather large module with 1388 genes. GO analysis showed enrichment for cilium related genes (Table 3), while the hub genes were enriched for genes involved in extracellular matrix organization, e.g. *CYR61*, *CSGALNACT1*, *ANXA2*, *AGT*, *COL21A1*, *EFEMP1* and *ECM2* (Additional file 4D). The highly negatively correlated CNneg1 module was enriched for genes involved in neuronal function, especially for genes involved in synaptic function/plasticity and ion channels (Table 3). This finding was reflected in the hub gene analysis of the CNneg1 module, in which about 50% of the identified hub genes are implicated to play a role in synaptic function (Additional file 4I). Furthermore, all CNneg1 hub genes were statistically significantly dysregulated as determined by Hodges and co-workers. The CNneg2 module represented the negatively correlated gene clusters, like e.g. mitochondrial and proteasomal genes, which we also observed in cerebellum and frontal cortex (Tables 1, 2, 3 and Figure 4E). In addition, this module was enriched for chaperone and spliceosome genes and genes required for DNA repair and translation initiation (Table 3). Consequently, also its hub genes were mostly enriched for mitochondrial genes (Figure 3F). Modules that were positively correlated with HD in the caudate nucleus network were, amongst others, enriched for transcriptional regulators, chromatin modifiers and genes involved in mRNA processing (modules CNpos1 and CNpos2, Table 3). Especially genes functioning in the development of blood vessel, glial cells, epithelial cells and astrocytes clustered in the hub genes of the CNpos2 module (Additional file 4A). Also, all hub genes in the CNpos1 and CNpos2 modules were statistically significantly dysregulated [27] (Figure 3E and Additional file 4A). Hub gene and GO analysis showed a very high enrichment for inflammatory response/immune system genes in the CNpos6 and CNpos8 modules (Table 3 and Additional file 4E and G). Noteworthy, 6 complement genes (*C1R, C1S, C1QA, C1QB, C3* and *C5AR1*) were assigned hub gene status in these modules including the central component C3 (Additional file 4G). However, only *C3* was previously found to be significantly dysregulated (Additional file 4E and G). The caudate nucleus network fit the analysis by Hodges and colleagues very well, as in total only 193 (5.0% of 3825 genes) of the significantly dysregulated genes were not sorted into modules that were correlated with HD.

**Table 3:** **Gene ontology enrichment for the caudate nucleus network**

**Module**	**cor**	**GO-term (DAVID)**	**Potential regulators**
CNpos1	up	regulation of transcription (6.18, 0.000)	YY1 (0.000)^2^, ELK1 (0.001)^2^, GABPB1 (0.002)^2^, SP1 (0.002)^2^, NRF1 (0.002)^2^, E2F (0.007)^2^, IRF1 (0.011)^2^, GTF3A (0.032)^2^, SOX9 (0.033)^2^
		chromatin modification (3.85, 0.003)	
		mRNA processing (3.71, 0.004)	
CNpos2	up	regulation of transcription (5.94, 0.001)	NFAT (0.004)^2^
		cell migration (4.09, 0.004)	
		lipid metabolism (2.51, 0.001)	
CNpos3	up	RNA binding (0.65, 1.0)	
CNpos4	up	chromatin organization (2.45, 0.008)	EGR2 (0.010)^2^, MYC (0.040)^2^, TCF3 (0.040)^2^, NR2F2 (0.040)^2^, TCF12 (0.040)^2^, SP1 (0.040)^2^, EGR1 (0.040)^2^, EGR4 (0.040)^2^
CNpos5	up	cilium (2.79, 0.003)	
CNpos6	up	inflammatory response (8.33, 0.000)	STAT5A (0.000)^2^, STAT3 (0.000)^2^, STAT5B (0.000)^2^, BACH2 (0.002)^2^, NFAT (0.005)^2^, JUN (0.020)^2^, NFE2 (0.035)^2^
CNpos7	up	regulation of transcription (3.0, 0.045)	
CNpos8	up	inflammatory response (14.15, 0.000)	ELF1 (0.000)^2^, STAT1/STAT2 (0.017)^2^, IRF1 (0.017)^2^
		icosanoid metabolism (1.73, 0.001)	
CNpos9	up	myelination (3.06, 0.002)	
		oligodendrocyte/glial differentiation (2.4, 0.054)	
CNneg1	down	synapse (12.23, 0.000)	miR16 (0.018)^1^, NRF1 (0.03)^1^ REST (0.000)^2^, EGR1 (0.000)^2^, SF1 (0.000)^2^, CREB1 (0.000)^2^, JUN (0.000)^2^, EGR4 (0.000)^2^, MYOD1 (0.000)^2^, TCF3 (0.000)^2^, RORA (0.000)^2^, ATF1 (0.000)^2^, E4F1 (0.000)^2^, SP1 (0.000)^2^, ESRRA (0.001)^2^, TCF11 (0.002)^2^, PAX4 (0.002)^2^, TFAP4 (0.003)^2^, MAZ (0.003)^2^, HAND1 (0.006)^2^, EGR2 (0.007)^2^, NRF2 (0.007)^2^, ATF3 (0.008)^2^, RFX1 (0.008)^2^, POU3F1 (0.008)^2^, LEF1 (0.011)^2^, POU1F1 (0.019)^2^, MYB (0.027)^2^, TCF12 (0.041)^2^, NFE2 (0.041)^2^, MEIS1 (0.044)^2^, SREBF1 (0.050)^2^
		ion channels (4.61, 0.000)	
		regulation of synaptic plasticity (4.58, 0.000)	
		protein transport (2.89, 0.011)	
		protein targeting to mitochondrion (2.75, 0.007)	
CNneg2	down	mitochondrion (20.31, 0.000)	YY1 (0.005)^1^, ETS1 (0.005)^1^, NRF1 (0.005)^1^ ELK1 (0.000)^2^
		proteasome/protein catabolic process (5.83, 0.000)	
		mitochondrial ribosome (4.54, 0.000)	
		chaperones (3.17, 0.012)	
		spliceosome (3.0, 0.002)	
		DNA repair (2.47, 0.027)	
		translation initiation (1.95, 0.007)	
CNneg3	down	hemoglobin complex (1.74, 0.024)	
HTT	down	neuron projection (1.93, 0.56)	

Gene ontology (GO) enrichment for the caudate nucleus network. Genes in the identified modules were analyzed using DAVID. The sign of the correlation (cor) with HD and the over-represented GO-terms are shown. The first number in brackets after the GO-term is the respective fold enrichment, the second number the adjusted *P*-value, as determined by DAVID. All significantly enriched (adjusted *P* <0.05) GO-terms are shown. In cases where no significantly enriched GO-term was identified, the GO-term with the highest fold enrichment is shown. Potential regulators of a module were identified using ^1^GO-Elite, or ^2^WebGestalt. Adjusted *P*-values are given in brackets after the name. Regulators that were identified by both tools are highlighted in bold. HTT is part of the CNneg1 module in the caudate nucleus network. The GO-term enrichment for 100 genes with the highest correlation with HTT is shown.

### Comparison of the three human brain region networks

To investigate the similarity of transcriptional dysregulation between tissues, we compared the significantly positively and negatively correlated genes in the three networks (Figure 4A), as well as the conservation of hub genes (Figure 4B-F). Using the caudate nucleus network as the basis, we found that both cerebellum and frontal cortex BA4 networks exhibited considerable similarities of significantly correlated genes (Figure 4A). More than 1200 genes were correlated in the same way in all three brain regions; the correlations of 1563 genes were conserved between caudate nucleus and frontal cortex BA4 and 1724 genes were similarly correlated in caudate nucleus and cerebellum (Figure 4A). Gene ontology enrichment analysis showed that metallothioneins and genes involved in the stress response and angiogenesis were commonly positively correlated with HD in all three networks (Table 4). Genes implicated in mitochondrial function, glycolysis, intracellular protein transport, proteasome and synaptic vesicles were commonly negatively correlated with HD in all three networks (Table 4). Furthermore, we found extensive conservation of hub genes in modules that were positively correlated with HD (Figure 4B to D). However, only one set of modules, which were negatively correlated with HD, exhibited common hub genes (Figure 4E). Interestingly, the CNneg1 module of the caudate nucleus network, which represented the “neuron/synaptic” module had only 7 hub genes overlap within several cerebellum modules and only 3 hub genes overlap in the frontal cortex BA4 region network, indicating its tissue specific character (data not shown). The CBpos5 cerebellum module and the FC4pos4 frontal cortex BA4 module were both enriched for chaperone genes (Tables 1 and 2). Consequently, we identified many chaperone genes, which had hub gene status in both networks (Figure 4F).

**Table 4:** **Gene ontology enrichment for conserved genes between HD networks**

**Overlap**	**GO-term (DAVID)**	**Potential regulators**
**Positively correlated genes**		
CN and FC-BA4	inflammatory response (5.6, 0.001)	MYC/MAX (0.010)^2^, STAT3 (0.010)^2^, ETS2 (0.020)^2^
	epithelial to mesenchymal transition (1.18, 0.026)	
CN and CB	regulation of transcription (5.2, 0.000)	
	mRNA processing (3.69, 0.001)	
	apical junction complex (1.91, 0.027)	
FC-BA4 and CB	zinc-finger (1.17, 0.44)	
all three networks	metallothionein (5.1, 0.000)	FOXF2 (0.002)^2^, NFIL3 (0.010)^2^, LEF1 (0.017)^2^, HSF1 (0.017)^2^, ATF2 (0.022)^2^, HIF1A (0.026)^2^, SP1 (0.042)^2^
	stress response/chaperones (2.62, 0.02)	
	angiogenesis (2.61, 0.039)	
**Negatively correlated genes**		
CN and FC-BA4	synaptic transmission (4.8, 0.000)	REST (0.004)^2^, EGR4 (0.015)^2^, SP1 (0.015)^2^, ATF1 (0.022)^2^, MEIS1 (0.022)^2^, ELK1 (0.022)^2^, ESRRA (0.040)^2^, ATF3 (0.046)^2^, E4F (0.046)^2^, SF1 (0.046)^2^, LEF1 (0.046)^2^
	ion channels (4.65, 0.000)	
	protein catabolic process (4.23, 0.008)	
CN and CB	mitochondrion (8.46, 0.000)	SF1 (0.002)^2^, E4F (0.020)^2^
	intracellular protein transport (3.02, 0.023)	
	vesicle mediated transport (2.19, 0.013)	
FC-BA4 and CB	coenzyme metabolic process (1.86, 0.98)	ELK1 (0.007)^2^, E4F (0.007)^2^
all three networks	mitochondrion (6.01, 0.000)	CREB (0.000)^2^, ATF3 (0.001)^2^, SF1 (0.015)^2^, ERR1 (0.015)^2^, TCF11 (0.015)^2^, ELK1 (0.018)^2^, ATF4 (0.018)^2^, SREBF1 (0.018)^2^, ATF6 (0.019)^2^, E4F (0.024)^2^, JUN (0.026)^2^, EGR1 (0.039)^2^, NRF1 (0.049)^2^
	glycolysis (2.74, 0.003)	
	intracellular protein transport (2.59, 0.028)	
	proteasome (1.97, 0.001)	
	synaptic vesicle (1.82, 0.018)	

Gene ontology (GO) enrichment for conserved genes between HD networks. Genes were analyzed using DAVID and the over-represented GO-terms are shown. The first number in brackets after the GO-term is the respective fold enrichment, the second number the adjusted *P*-value, as determined by DAVID. All significantly enriched (adjusted *P* <0.05) GO-terms are shown. In cases where no significantly enriched GO-term was identified, the GO-term with the highest fold enrichment is shown. Potential regulators of a module were identified using ^1^GO-Elite, or ^2^WebGestalt. Adjusted *P*-values are given in brackets after the name.

### Meta analysis of the caudate nucleus network with other disorders

**Figure 5:**
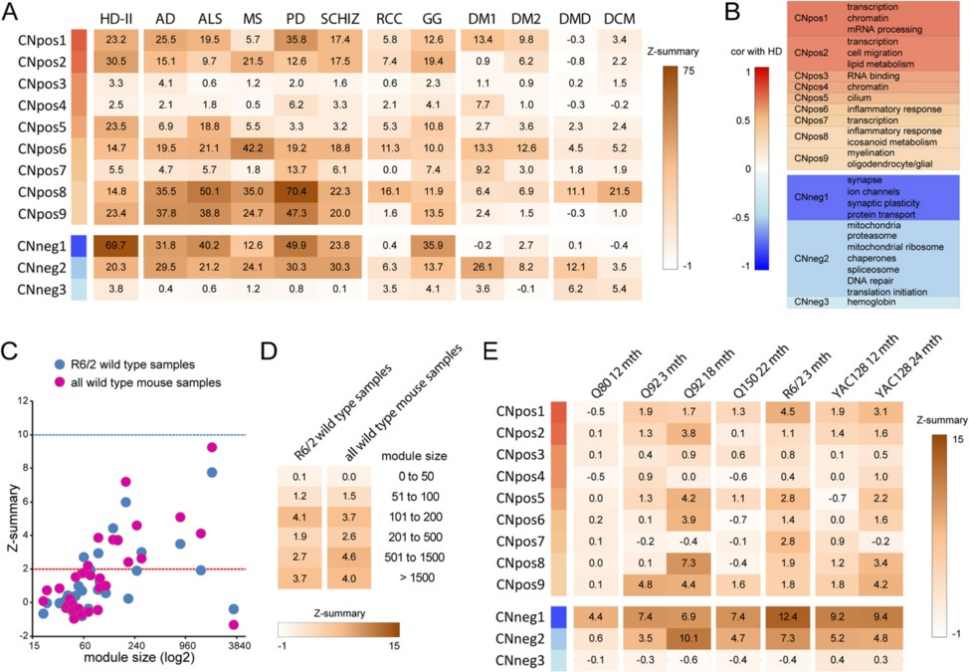
**Preservation analysis of HD caudate nucleus network modules in other diseases and in HD mouse models highlights common transcriptional changes. (A)** Preservation analysis of HD caudate nucleus network modules in various diseases. The Z-summary values are shown as a heat map from white (−1) to brown (75). Colors next to the modules indicate correlation (cor) with HD, as shown in **(B)**, together with a short summary table of Table 3. HD-II = Huntington’s disease dataset 2; AD = Alzheimer’s disease; ALS = Amyotrophic lateral sclerosis; MS = multiple sclerosis; PD = Parkinson’s disease; SCHIZ = Schizophrenia; RCC = renal cell carcinoma; GG = ganglioglioma; DM1, DM2 = myotonic dystrophy type 1, type 2; DMD = Duchenne Muscular Dystrophy; DCM = dilated cardiomyopathy. For details of the datasets see Table 5. **(C and** **D)** A HD caudate nucleus network with only control samples as the input for the preservation analysis was generated (n = 32). **(C)** Preservation analysis of these human caudate modules in a dataset of only wild type mouse samples from the R6/2 dataset (n = 9), or all wild type mouse samples, respectively (n = 22) (excluding the Q80 data, due to the different type of microarray). **(D)** The median Z-summary values from the analysis in **(C)** for modules of certain size ranges were calculated and are shown as a heat map from white (−1) to brown (15). **(E)** Preservation analysis of HD caudate nucleus network modules in HD mouse models. The Z-summary values are shown as a heat map from white (−1) to brown (15). Colors next to the modules indicate correlation with HD, as shown in **(B)**. Q80 = *Hdh*4^80Q^; Q92 = *Hdh* ^Q92^; Q150 = *Hdh*Q150; mth = months.

Next, we analyzed the preservation of the caudate nucleus network modules in gene expression datasets of other disorders (Figure 5). We used our caudate nucleus network, which was derived from the most affected brain tissue in HD and compared it to other highly affected tissues (Table 5). In addition to other neurodegenerative disorders (Alzheimer’s disease (AD), amyotrophic lateral sclerosis (ALS), multiple sclerosis (MS), Parkinson’s disease (PD) and schizophrenia (SCHIZ)), we also included muscle related diseases (myotonic dystrophy type 1/type 2 (DM1, DM2) and Duchenne Muscular Dystrophy (DMD)), dilated cardiomyopathy (DCM) and cancers (renal cell carcinoma (RCC) and ganglioglioma (GG)). When we analyzed the preservation of the caudate nucleus modules [27] in another HD dataset (HD-II) [35], we observed very high Z-summary scores for most modules, indicating a good reproducibility and thus robustness of the HD networks. The CNpos3 and CNpos4 modules were only assigned a few genes (Figure 1F), which most probably was the reason for their low preservation score (Figure 5A). The CNpos7 and CNneg3 modules had an average number of genes assigned to them. However, they appeared to be rather dataset specific, as we generally observed low Z-summary scores (Figure 5A). Other diseases that result in pronounced neurodegeneration, e.g. AD, ALS, MS, PD, SCHIZ or GG also exhibited high preservation scores for most modules. As controls for our preservation analysis we used the RCC, DMD and DCM data. In these datasets, Z-summary scores were low, apart from the two “inflammatory” modules CNpos6 and CNpos8, which were highly preserved in virtually all datasets (Figure 5A and B). Interestingly, the highest correlated (with HD) modules CNpos1 and CNneg1 showed only moderate preservation in MS. However, the two “inflammatory” modules again were very highly preserved between the HD and MS datasets (Figure 5A). For the muscle wasting disease myotonic dystrophy, type 1 showed higher preservation scores than type 2. But notably both were characterized by a lack of preservation of the CNneg1 “neuron/synaptic” module, yet high preservation of the CNneg2 and CNpos1 modules (Figure 5A and B). This finding once more highlights the aforementioned successful separation of the synaptic/neuronal (CNneg1) transcriptional dysregulation from the more ubiquitous dysregulated genes (CNneg2) in our networks.

**Table 5:** **Microarray datasets used in this study**

**Set**	**Accession**	**ctr/patient**	**Tissue**	**Array**	**Overlap**	**Reference**
main HD	GSE3790	26/38	cerebellum	GPL96	100%	[27]
		16/18	BA4 region of frontal cortex			
		12/18	BA9 region of frontal cortex			
		32/36	caudate nucleus			
AD	GSE26927	7/11	entorhinal cortex	GPL6255	94.2%	[35]
ALS		9/10	cervical spinal cord			
HD-II		10/9	ventral head of the caudate nucleus			
MS		10/8	superior frontal gyri			
PD		8/12	substantia nigra			
SCHIZ		8/9	temporal cortex left, BA22 region			
DM1	GSE7014	5/10	skeletal muscle	GPL570	100%	[81]
DM2		5/20	skeletal muscle			
DMD	GSE6011	14/22	quadriceps muscle	GPL96	100%	[82]
DCM	GSE3585	5/7	heart	GPL96	100%	[83]
RCC	GSE781	5/12	kidney	GPL96	100%	[84]
GG	E-MEXP-1690	6/6	brain	GPL96	100%	[85]
**Set**	**Accession**	**WT/HD**	**Tissue**	**Array**	**Overlap**	**Reference**
Q80	GSE10263	3/3	striatum	GPL81	51.2%	[28]
Q150	GSE10263	4/4	striatum	GPL1261	81.9%	
Q92	GSE7958	3/3 (3 mth)	striatum	GPL1261	81.9%	
		3/3 (18 mth)				
R6/2	GSE10263	9/9	striatum	GPL1261	81.9%	
YAC128	GSE19677	4/4 (12 mth) 3/6 (24 mth)	striatum	GPL1261	81.9%	[24]

The abbreviations for the datasets are as follows: main HD = main Huntington’s disease dataset; AD = Alzheimer’s disease; ALS = Amyotrophic lateral sclerosis; HD-II = Huntington’s disease dataset 2; MS = multiple sclerosis; PD = Parkinson’s disease; SCHIZ = schizophrenia; DM1, DM2 = myotonic dystrophy type 1, type 2; DMD = Duchenne muscular dystrophy; DCM = dilated cardiomyopathy; RCC = renal cell carcinoma; GG = ganglioglioma; Q80 = *Hdh*4^80Q^; Q150 = *Hdh*Q150; Q92 = *Hdh* ^Q92^. Accession is the accession number of the EMBL-EBI ArrayExpress, or NCBI Gene Expression Omnibus (GEO). Ctr/patient and WT/tg gives the sample numbers after outlier removal for control (ctr) or patient samples and wild type (WT) or HD mouse model samples (HD), respectively. For details of outlier removal procedure see materials and methods. Array lists the microarray type used for the particular study; for details see the GEO database. Overlap gives the percentage of genes, which are detected on the particular chip in comparison to the main HD array (GPL96).

### Meta analysis of the caudate nucleus network with mouse models of HD

Many models of HD exist, amongst which the murine models are the best studied [36,37]. However only limited data on transcriptome wide dysregulation is publicly available and the cross-species comparison additionally complicates meta analyses. To gain a first impression how the species comparison might affect the preservation analysis we compared only the caudate nucleus samples from control subjects with the wild type striatum mouse samples. To this end, we computed a network for the human control samples and calculated the preservation Z-summary score for its modules in the R6/2 wild type samples, or all mouse wild type data except the Q80 dataset, respectively (Figure 5C and D). We only included genes that were conserved between both species. Both meta analyses of control human caudate nucleus and wild type mouse striatum data were very comparable and we obtained median Z-summary values of less than 5 for the preservation of modules of defined sizes (Figure 5C and D). This led us to hypothesize that Z-summary values above this value would indicate high cross-species preservation and would be correlated to effects induced by mutant huntingtin. The Q80 dataset was obtained with a different type of microarray than the other mouse datasets (Table 5), in which only about 50% of the genes were conserved and therefore Z-summary values were very low (Figure 5E). In general, for the other mouse datasets, preservation was better in fully symptomatic animals (Figure 5E, compare time points for Q92 and YAC128), with absolute age of the animals being irrelevant. Given our data for the control/wild type cross-species comparison (Figure 5C and D), the negatively correlated modules of the human HD caudate nucleus dataset were very highly preserved in the mouse models, while the positively correlated modules were in general less highly preserved (Figure 5E).

**Figure 6:**
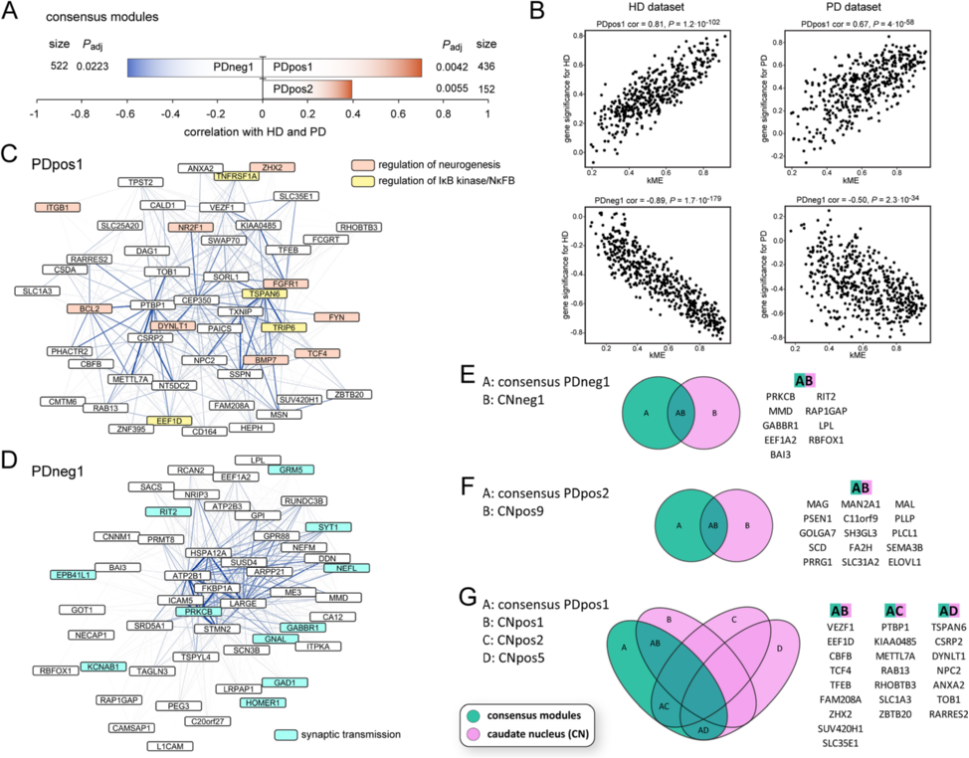
**WGCNA analysis of the HD/PD consensus dataset indicates commonly dysregulated pathways. (A)** Visualization of modules that are highly correlated with Huntington’s (HD) and Parkinson’s (PD) disease state. Size is the number of genes for each module. *P*_adj_ gives the Benjamini Hochberg corrected significance value of correlation with HD/PD for each module. **(B)** Correlations of eigengene based connectivity (kME) versus the gene significance for HD and PD. The two modules with the highest absolute correlation are shown for each disease dataset. cor = correlation. **(C and** **D)** Visualization of hub genes in HD/PD consensus network modules. The 50 most connected genes (nodes) and the 500 strongest gene-gene interactions (edges) in each module are shown. The width and the color saturation of the lines (edges) correspond to the weight of the interactions. The PDpos2 module is visualized in Additional file 5. **(E -** **G)** Hub gene comparison of HD/PD consensus modules versus modules of the HD caudate nucleus (CN) dataset. Venn diagrams show the overlap of hub genes in the respective consensus modules with HD caudate nucleus modules. Only consensus modules with an overlap of 5 or more genes to CN modules are shown. For analysis of a HD/GG consensus network see Additional file 6; HD/RCC see Additional file 7; HD/DM1 see Additional file 8; HD/DM2 see Additional file 9.

**Figure 7:**
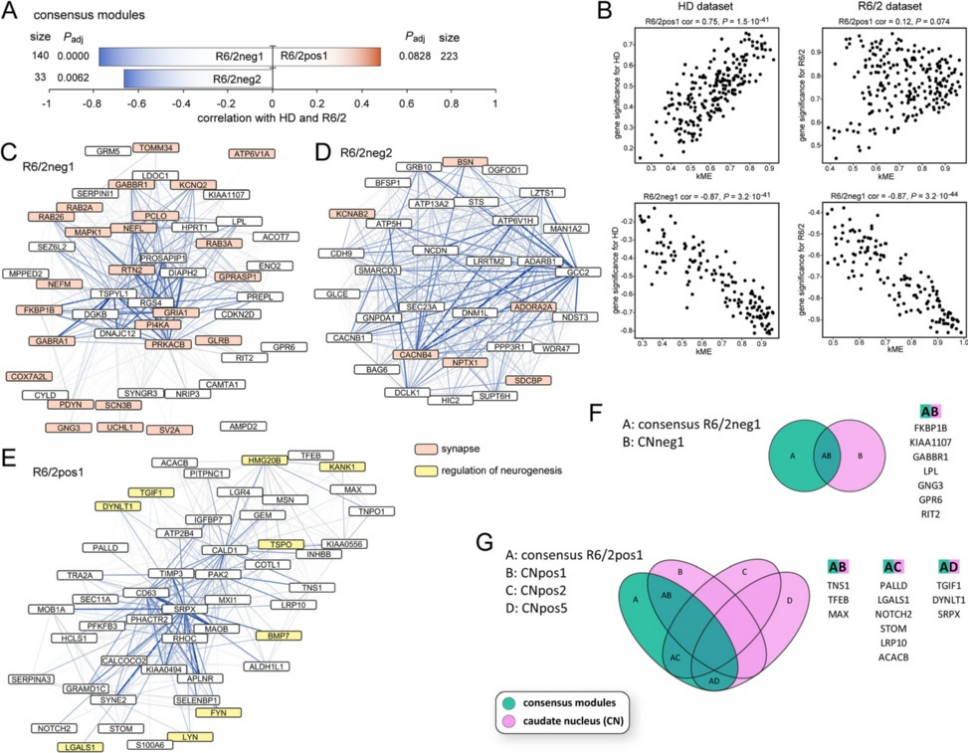
**WGCNA analysis of the human HD/R6/2 consensus dataset indicates commonly dysregulated pathways. (A)** Visualization of modules that are highly correlated with Huntington’s disease (HD) state and genotype of R6/2 mice. Size is the number of genes for each module. *P*_adj_ gives the Benjamini Hochberg corrected significance value of correlation with human HD/R6/2 for each module. **(B)** Correlations of eigengene based connectivity (kME) versus the gene significance for human HD and R6/2. The two modules with the highest absolute correlation are shown for each dataset. cor = correlation. **(C -** **E)** Visualization of hub genes in human HD/R6/2 consensus network modules. The 50 most connected genes (nodes) and the 500 strongest gene-gene interactions (edges) in each module are shown. The width and the color saturation of the lines (edges) correspond to the weight of the interactions. **(F and** **G)** Hub gene comparison of human HD/R6/2 consensus modules versus modules of the HD caudate nucleus (CN) dataset. Venn diagrams show the overlap of hub genes in the respective consensus modules with HD caudate nucleus modules. Only consensus modules with an overlap of 5 or more genes to CN modules are shown.

**Figure 8:**
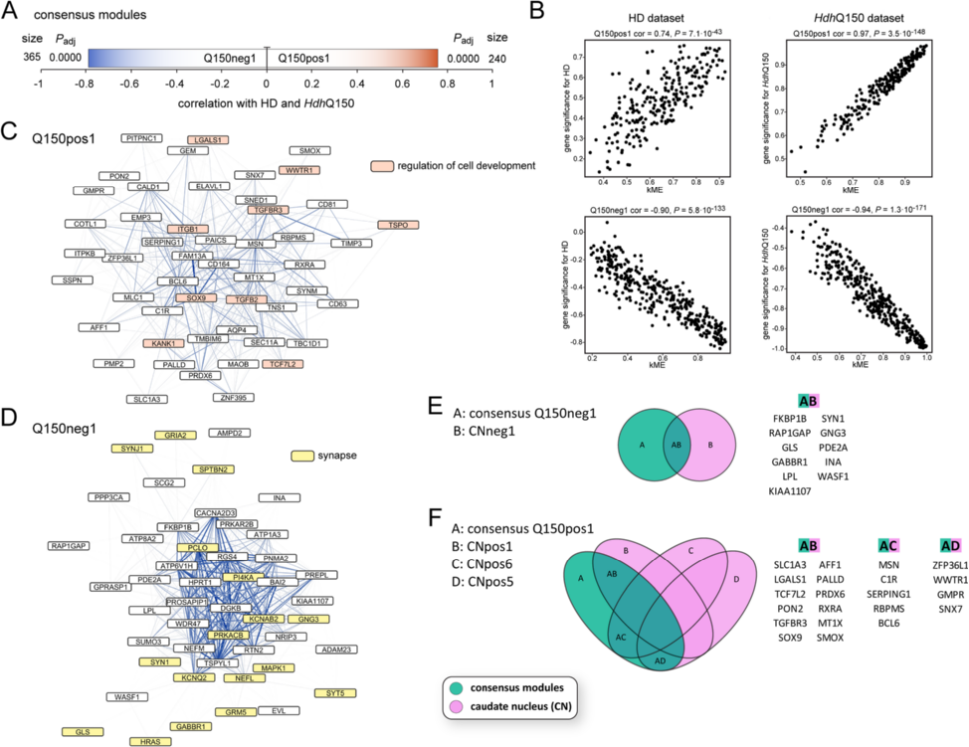
**WGCNA analysis of the human HD/** ***Hdh*** **Q150 consensus dataset indicates commonly dysregulated pathways. (A)** Visualization of modules that are highly correlated with Huntington’s disease (HD) state and genotype of *Hdh*Q150 mice. Size is the number of genes for each module. *P*_adj_ gives the Benjamini Hochberg corrected significance value of correlation with human HD/*Hdh*Q150 for each module. **(B)** Correlations of eigengene based connectivity (kME) versus the gene significance for human HD and *Hdh*Q150. The two modules with the highest absolute correlation are shown for each dataset. cor = correlation. **(C and** **D)** Visualization of hub genes in human HD/*Hdh*Q150 consensus network modules. The 50 most connected genes (nodes) and the 500 strongest gene-gene interactions (edges) in each module are shown. The width and the color saturation of the lines (edges) correspond to the weight of the interactions. **(E and** **F)** Hub gene comparison of human HD/*Hdh*Q150 consensus modules versus modules of the HD caudate nucleus (CN) dataset. Venn diagrams show the overlap of hub genes in the respective consensus modules with HD caudate nucleus modules.

To reveal further similarities between gene expression in the human HD samples and other disorders or the mouse models, respectively, we computed consensus networks. In this type of network, only genes, which are similarly regulated in both disorders or species and which are assigned to modules that are correlated with the trait of interest in both datasets, are analyzed. In our preservation analysis we observed very high Z-summary values for the PD dataset (Figure 5A). Accordingly, we also identified consensus modules, which were highly correlated both with HD and PD disease states (Figure 6A and B). Gene ontology enrichment and hub gene analysis showed that these modules, which were positively correlated with HD and PD, were enriched in genes implicated in NFκB signaling, neurogenesis and lipid synthesis (Figure 6C, Table 6 and Additional file 5). The negatively correlated module in the HD/PD consensus network was enriched for genes involved in synaptic and mitochondrial function, as well as calmodulin binding proteins (Figure 6D and Table 6). In addition, there was a high overlap of hub genes of consensus network modules with the hub genes that we identified in the caudate nucleus dataset (Figure 6E-G). Interestingly, several Alzheimer’s disease related genes (*PSEN1, SORL1, FGFR1, BMP7, FYN, BCL2, SCD, NPC2, PTBP1, TNFRSF1A, ITGB1, LHPP, LRP2, LRPAP1, LPL, L1CAM, CNNM1 and HSPA12A*) had hub gene status in this HD/PD consensus network (Figure 6E-G). Next, we analyzed consensus networks of the HD caudate nucleus dataset with the datasets of other disorders, the same we had used in the preservation analysis. We did not find significantly correlated modules (with disease) in the consensus networks for HD with AD, ALS, MS and SCHIZ. However, we could identify significantly correlated modules with the two cancer datasets (Additional files 6 and 7) and the two types of myotonic dystrophies (Additional file 8 and 9). Taken together, in these consensus networks, the negatively correlated modules were mostly enriched for genes involved in neuronal and mitochondrial function, the positively correlated modules were mostly enriched for inflammatory pathway genes in the two cancers and for regulators of transcription and neurogenesis in DM1 and DM2 (Additional files 6, 7, 8 and 9 and Table 6). This largely mirrored the preservation analysis (Figure 5). We also could identify significantly correlated modules in the R6/2 and *Hdh*Q150 datasets (Figures 7 and 8). The negatively correlated modules for both mouse models were enriched for synaptic genes (Figures 7C and D, 8D and Table 7) and many of these also had hub genes status in the human CNneg1 module (Figures 7F and 8E). GO analysis of the positively correlated module in the human HD/R6/2 consensus network showed genes involved in lipid metabolism and regulation of neurogenesis (Figure 7E and Table 7). In the positively correlated module of the human HD/*Hdh*Q150 consensus network, we found an enrichment for extracellular matrix genes and regulators of cell development (Figure 8C and Table 7). Furthermore, the positively correlated modules for both mouse models exhibited extensive overlap of hub genes with positively correlated modules in the human caudate nucleus dataset (Figures 7G and 8F).

**Table 6:** **Gene ontology enrichment for the consensus networks in human datasets**

**Module**	**cor**	**GO-term (DAVID)**	**Potential regulators**
**Parkinson’s disease (PD)**			
PDpos1	up	IκB kinase/NFκB (3.59, 0.032)	
PDpos2	up	lipid synthesis (2, 0.01)	
PDneg1	down	synapse (5.87, 0.000)	miR16 (0.036)^1^
		mitochondrion (4.6, 0.000)	ESRRA (0.001)^2^, SF1 (0.003)^2^
		calmodulin binding (3.12, 0.044)	
**Myotonic dystrophy type 1 (DM1)**			
DM1pos1	up	regulation of neurogenesis (1.55, 0.99)	MEF2 (0.040)^2^, E2F (0.040)^2^, NR3C1 (0.040)^2^, PITX2 (0.040)^2^, ATF6 (0.040)^2^, VDR (0.040)^2^, ATF1 (0.040)^2^, TP53 (0.041)^2^
DM1neg1	down	axon (1.18, 0.98)	POU2F1 (0.011)^2^, POU1F1 (0.034)^2^, IRF2 (0.048)^2^
DM1neg2	down	enzyme activator activity (2.52, 0.049)	
DM1neg3	down	synapse (2.29, 0.008)	SF1 (0.001)^2^, REST (0.032)^2^
**Myotonic dystrophy type 2 (DM2)**			
DM2pos1	up	lysosome (3.03, 0.043)	
DM2pos2	up	regulation of transcription (1.51, 0.99)	
DM2pos3	up	tubulin binding (1.64, 0.52)	
DM2pos4	up	sarcomer (1, 1.0)	
DM2neg1	down	mitochondrion (3.09, 0.01)	
DM2neg2	down	dendrite (1.91, 0.51)	NRF1 (0.009)^1^, ETS1 (0.054)^1^
**Ganglioglioma (GG)**			
GGpos1	up	inflammatory response (6.32, 0.002)	NFκB (0.022)^1^, miR124 (0.022)^1^, miR106b (0.022)^1^, MYOG (0.066)^1^
		cell adhesion/extracellular matrix (4.13, 0.002)	ELF1 (0.000)^2^, IRF8 (0.008)^2^, MYB (0.008)^2^, ELK1 (0.010)^2^, SPI1 (0.010)^2^, CEBPA (0.016)^2^, NFAT (0.029)^2^, IRF1 (0.034)^2^, STAT5A (0.034)^2^, AHR (0.043)^2^, SOX5 (0.049)^2^, TP53 (0.049)^2^
GGneg1	down	axon (11.88, 0.000)	REST (0.000)^2^, SF1 (0.000)^2^, TCF3 (0.000)^2^, ESRRA (0.000)^2^, MYOD (0.000)^2^, RFX1 (0.000)^2^, RORA (0.000)^2^, EGR1 (0.000)^2^, JUN (0.000)^2^, TCF11 (0.000)^2^, ATF3 (0.000)^2^, LEF1 (0.000)^2^, PAX4 (0.000)^2^, E4F1 (0.000)^2^, CREB (0.000)^2^, HLF (0.001)^2^, MAZ (0.001)^2^, SP1 (0.001)^2^, NFIL3 (0.001)^2^, BACH1 (0.002)^2^, ATF2 (0.002)^2^, ATF1 (0.002)^2^, TFAP4 (0.002)^2^, TCF8 (0.003)^2^, ZNF238 (0.004)^2^, NFE2 (0.005)^2^, HSF1 (0.006)^2^, MIF (0.010)^2^, CUTL1 (0.012)^2^, SREBF1 (0.016)^2^, NF1 (0.020)^2^, MEIS1 (0.020)^2^, HSF2 (0.021)^2^, NFE2L2 (0.021)^2^, PCAF (0.023)^2^, GCF1 (0.034)^2^, ITGAL (0.034)^2^, ATF4 (0.035)^2^, MAF (0.038)^2^, TAL1 (0.043)^2^, NR1H4 (0.044)^2^, GATA2 (0.044)^2^, SOX9 (0.046)^2^
		synapse (11.64, 0.000)	
		microtubuli based transport (5.18, 0.000)	
		calmodulin binding (4.87, 0.000)	
		cytoskeleton (3.63, 0.000)	
		neuropeptide (1.96, 0.012)	
		signaling from G-protein families (1.49, 0.025)	
**Renal cell carcinoma (RCC)**			
RCCpos1	up	inflammatory response (12.47, 0.000)	NFκB (0.06)^1^, **E2F** (0.081)^1^
		regulation of IκB kinase/NFκB (5.54, 0.000)	IRF8 (0.000)^2^, IRF1 (0.000)^2^, ETS2 (0.000)^2^, ELF1 (0.000)^2^, SPI1 (0.000)^2^, ELF2 (0.001)^2^, STAT1 (0.001)^2^, **E2F** (0.001)^2^, ETS1 (0.007)^2^, GABPA (0.007)^2^, ELK1 (0.010)^2^, STAT5B (0.021)^2^, FOXO4 (0.022)^2^, TP53 (0.024)^2^, AHR (0.025)^2^, ETV4 (0.026)^2^, STAT3 (0.032)^2^, SMAD1 (0.037)^2^, IRF7 (0.037)^2^, AR (0.040)^2^
		angiogenesis (5.17, 0.004)	
		caspase recruitment (4.8, 0.002)	
		regulation of transcription (4.03, 0.003)	
		regulation of apoptosis (3.09, 0.004)	
		extracellular matrix (2.81, 0.005)	
		chromatin (2.6, 0.023)	
RCCpos2	up	semaphorin/CD100 antigen (1.45, 0.023)	
RCCneg1	down	mitochondrion (31.99, 0.000)	CREB (0.006)^1^, NRF1 (0.006)^1^, miR16 (0.068)^1^
		protein catabolic process/proteasome (2.29, 0.037)	SF1 (0.000)^2^, ESRRA (0.000)^2^, E4F1 (0.006)^2^, JUN (0.030)^2^, ATF3 (0.030)^2^, NRF1 (0.047)^2^
		synaptic vesicle (1.34, 0.029)	

Gene ontology (GO) enrichment for the consensus network analysis of the HD caudate nucleus dataset with various diseases. Genes in the identified modules were analyzed using DAVID. The sign of the correlation (cor) and the over-represented GO-terms are shown. The first number in brackets after the GO-term is the respective fold enrichment, the second number the adjusted *P*-value, as determined by DAVID. All significantly enriched (adjusted *P* <0.05) GO-terms are shown. In cases where no significantly enriched GO-term was identified, the GO-term with the highest fold enrichment is shown. Potential regulators of a module were identified using ^1^GO-Elite, or ^2^WebGestalt. Adjusted *P*-values are given in brackets after the name. Regulators that were identified by both tools are highlighted in bold.

**Table 7:** **Gene ontology enrichment for the consensus networks in mouse datasets**

**Module**	**cor**	**GO-term (DAVID)**	**Transcription factor**
***Hdh*** **Q150**			
Q150pos1	up	extracellular matrix (1.71, 0.65)	
Q150neg1	down	synaptic transmission/synapse (8.81, 0.000)	CREB (0.045)^1^
		neuron projection/axon (2.4, 0.019)	
		mitochondrion (2.1, 0.007)	
**R6/2**			
R6/2pos1	up	fatty acid metabolism (1.98, 0.13)	SOX9 (0.049)^2^
R6/2neg1	down	synaptic transmission (6.77, 0.000)	
		gated channel activity (2.3, 0.02)	
		neurotransmitter transport (2.08, 0.005)	
R6/2neg2	down	ion transport (1.68, 0.64)	

Gene ontology (GO) enrichment for the consensus network analysis of the HD caudate nucleus dataset with HD mouse models. Genes in the identified modules were analyzed using DAVID. The sign of the correlation (cor) and the over-represented GO-terms are shown. The first number in brackets after the GO-term is the respective fold enrichment, the second number the adjusted *P*-value, as determined by DAVID. All significantly enriched (adjusted *P* <0.05) GO-terms are shown. In cases where no significantly enriched GO-term was identified, the GO-term with the highest fold enrichment is shown. Potential regulators of a module were identified using ^1^GO-Elite, or ^2^WebGestalt. Adjusted *P*-values are given in brackets after the name.

## Discussion

In this study we use weighted correlation networks to analyze gene expression data from different brain regions of HD patients and compared them to other disorders and HD mouse models. In summary, we found comparable dysregulation to previously reported changes [27] in the BA4 and BA9 regions of the frontal cortex and the caudate nucleus. However, in contrast to previous findings, our analysis of the cerebellum detected extensive transcriptional dysregulation, to a similar extent to that seen in the BA4 region of the frontal cortex. Subsequent in depth comparison of the brain region specific networks revealed an underlying common transcriptional signature in all three brain regions: a negative correlation with HD for mitochondrial function, glycolysis, intracellular protein transport, proteasome and synaptic vesicles and a positive correlation with HD for metallothioneins and genes involved in stress response pathways and angiogenesis. Moreover, meta analyses of the caudate nucleus network and other disorders showed similarities for these disorders with HD, in particular with a high enrichment for inflammatory pathway genes. Lastly, we compared well studied HD mouse models to the human gene expression dataset, which implied that whilst the mouse models mimic some aspects of the disease very well, certain aspects, for example induction of the inflammatory response, were only poorly reflected.

Yet, there are certain limitations and considerations for data interpretation of the current study. As with all *post mortem* tissues, RNA quality might be a problem. However, the authors of the original publication used rigorous standards to ensure comparable RNA quality and microarray reads [27]. In addition, we used a connectivity based outlier test to remove samples that didn’t pass our quality control [38] (see also materials and methods section). A further consideration is that the *post mortem* samples provide a snapshot of end stage disease gene expression, which might not necessarily reflect the underlying changes at or before disease onset or during disease progression. We consequently found the best preservation of human striatal gene expression changes in late stage mouse models (Figure 5E). Collection of patient samples, e.g. from peripheral tissues such as muscle at different disease stages might shed more light on the regulation of gene expression during disease progression. What’s more, the massive amount of neurodegeneration, greater than 90% in the striatum for grade 4 brains [39], poses the danger that changes in tissue/cell type composition are compared rather than changes in gene expression. Hodges and colleagues addressed this issue with laser-capture micro-dissection of control and patient tissue (grade 1) followed by the analysis of the same number of neurons for both. They found similar trends for gene expression changes in the captured neurons as compared to the tissue-based analysis and therefore, concluded that these changes occurred before cell death [27]. Our preservation and consensus network analyses showed that the mouse models very well mirror the “synaptic/neuronal” CNneg1 module of the human caudate nucleus network and in fact we observed the highest preservation scores for this module (Figure 5E). Furthermore, we found high enrichment for synaptic and neuronal genes in the negatively correlated modules in the R6/2 and *Hdh*Q150 consensus networks (Figures 7, 8 and Table 7). Given that very little striatal neurodegeneration occurs in both mouse models [40–42], the observed gene expression changes in the human datasets are most probably not due to differences in tissue composition and could therefore, at least partly, be the underlying cause for neuronal cell death. As for all bioinformatic predictions of dysregulated pathways *in vivo* validation is the logical next step. Regrettably, we do not have access to human HD brain tissue in sufficient quantity and quality to test the predictions of our analysis. For many, although not all of the predicted dysregulated pathways, there is an extensive literature that provides evidence for attenuated function in HD mouse models and HD patients (reviewed for example in [43,44]). Given that many of our predicted dysregulated pathways have been corroborated through the research reported by others, we expect that some of the novel predicted pathways will be confirmed in future studies as being affected in HD.

In a previous publication, Horvath and colleagues used a network based approach to compare the same HD patient caudate nucleus gene expression dataset to the modular structure of the transcriptome in normal human brains [30,38]. The focus of the study was however on the biologically meaningful relationship between samples, which can be distinguished with a connectivity based analysis. Nevertheless, they identified a module that had considerable similarity with the salmon “neuronal, synaptic and signal transduction” module of the normal human transcriptome and which was altered in HD. This is in very good agreement with our data, where we identified a homologous module (CNneg1) in the caudate nucleus network.

It is noteworthy to mention that a linear relationship between the correlation of a gene with disease and differential expression analysis does not necessarily exist. Therefore, although a gene can be highly correlated with a particular trait, the change in expression level can be relatively small. With standard dysregulation analysis, gene expression changes in a subpopulation of cells might be lost in a background of non-affected cells. With network analysis, we were able to find highly comparable GO enrichments and hub gene structures for the frontal cortex BA4 region even when analyzed together with the unaffected BA9 region. This implies that a weighted gene network approach can detect gene expression changes in a sub-population of cells, even against a huge background of expression signals of the same genes in unaffected tissue. The only major difference in analysis of the combined tissues was a reduction in the correlation of the modules with HD (Figures 1C and D, Table 3 and Additional files 10 and 11). We observed relatively small, yet statistically significant correlations of modules in the cerebellum with HD when compared to caudate nucleus, a finding, which is similar to that found in an HD mouse model [45]. One explanation for this effect could be that only a sub-population of cells in the cerebellum, yet to be determined, is affected.

In all three brain regions, we found genes involved in the function of mitochondria, glycolysis, intracellular protein transport, proteasome and synaptic vesicles to be commonly negatively correlated with HD, and metallothioneins and genes involved in stress response pathways and angiogenesis to be commonly positively correlated with HD (Table 4). This led us to hypothesize that the HD mutation causes a common transcriptional signature (Figure 5). Furthermore, the preservation of modules between each of the three brain region networks was generally very high, with only a few tissue specific modules (Figure 2). The CNpos5 module, specific to the caudate nucleus network, is potentially very interesting by virtue of its large size and correlation with HD (Figure 2E and F) and provides a good example of the drawbacks of gene ontology enrichment analysis. Historically driven, the gene ontology databases do not include the central nervous system specific functions of genes and are, as all large databases, plagued with incompletion, imprecision and a bias towards certain, well studied pathways [46]. So the only enriched functional cluster for this module contained genes implicated in cilium function (Table 3). Also, most probably because of the same aforementioned reasons, an upstream regulator was not identified. Yet, one of the advantages of connectivity based network analysis is the ranking of genes according to their co-regulation with other genes. This allows one to identify hub genes, which often are the biological key players in a particular module [47]. And indeed, 7 of the top 50 hub genes of the CNpos5 module have a probable function in extracellular matrix organization (*CSGALNACT1, CYR61, ANXA2, AGT, COL21A1, EFEMP1* and *ECM2*).

In addition to the newly identified transcriptional signature in the cerebellum, we found highly positively correlated modules enriched for genes involved in inflammation for both the cortex BA4 region and caudate nucleus (Tables 2 and 3). This is probably not surprising given the widespread gliosis that occurs in the brains of HD patients [48], however it was not identified in the original analysis [27]. A gene expression network study in late-onset AD identified an immune system/microglia module that was highly correlated with AD pathology [49]. In our comparison of HD with other disorders, the two “inflammatory” modules CNpos6 and CNpos8 were largely preserved in most disorders (Figure 5A) raising the possibility that treatments available for some of these could also be applied to HD [50–52]. Recently, mutant HTT was found to induce a cell-autonomous response in microglia [53] and astrocytes [54], which are first indications that neuroinflammation in HD is a potential contributing factor and not purely the consequence of neurodegeneration. Especially in the caudate nucleus network, we identified components of the complement system as being positively correlated with HD and many of these also had hub gene status (Additional file 4E and G). It has been previously reported that the complement system is abnormally activated in the brains of HD patients [55]. The complement system is regulated by the innate, as well as the adaptive immune system and is expressed in most cell types of the brain, thus it might be an important factor in neurodegenerative diseases [56]. Taken together, our data and previous findings suggest that targeting neuroinflammation, in particular activation of the complement system could be beneficial to slow down disease progression in HD.

Whether the abnormal folding of mutant HTT and/or the appearance of aggregates are sufficient to induce a cellular stress response, in particular through induction of steady state levels of molecular chaperones, is a long-standing question. To the best of our knowledge, our network analysis is the first indication that the expression of mutant HTT is correlated with higher levels of molecular chaperones in humans. We identified a significantly positively correlated module in the cerebellum network (CBpos5, Figures 1B and 3A, Table 1), which is highly enriched for protein folding/chaperone genes. We also found a similar module in the cortex BA4 network, that was just short of being significantly correlated (FC4pos4, Figure 1D, Table 2 and Additional file 3C). In contrast, we did not detect a similar module in the caudate nucleus network. It is tempting to speculate that the higher levels of these chaperones counteract the pathogenic effects of mutant HTT and protect these tissues (correlation *P*-value of chaperone modules CB < FC-BA4 < CN; pathogenesis CB < FC-BA4 < CN). We of course appreciate that further studies are needed, e.g. to investigate whether changes at the mRNA level are translated to changes at the protein level by assessing the protein levels of certain chaperones in different brain regions. In the frontal cortex BA4 region network we also identified a chaperone gene containing module that was negatively correlated with HD (FC4neg5, Table 2) highlighting the complex regulation of the proteostasis network and its potential suppression through other mechanism caused by mutant HTT. Together these data support the therapeutical avenue of drugs that boost the proteostasis network, which was shown numerous times in animal models to antagonize the progression of HD pathogenesis [57]. Given that the proteostasis networks in mammals does not seem to be impaired during aging [58], this approach might prove beneficial even in older patients.

As briefly addressed in the introduction, aberrant binding of an mRNA processing factor to the mutant *HTT* transcript results in the production of a HTT exon 1 fragment [19]. It might be expected that such an RNA toxicity based mechanism would have additional wide-spread consequences on global alternative splicing, similar to sequestration of MBNL1 in myotonic dystrophy. In fact, an unpublished study has identified various alternatively spliced transcripts in HD mouse model tissue (Gipson TA and Housman DE, unpublished data) and an increase of the 4R/3R tau mRNA ratio has been observed [59]. Intriguingly, we identified at least one module that was significantly enriched for genes involved in RNA binding/mRNA processing in all three brain region networks (Tables 1, 2 and 3), which had not been discovered in the original publication. In all cases the modules were positively correlated with HD indicative of an up-regulation of some parts of the RNA processing machinery. It will be very interesting to see in future studies, which splicing factors are mis-regulated and the consequences this has on general RNA processing in HD.

The similarities between HD and other neurodegenerative diseases point towards common pathogenic mechanisms (Figures 5, 6 and Additional files 6, 7, 8 and 9). Apart from the previously mentioned inflammatory component, we observed very high preservation scores for the CNneg2 module of the caudate nucleus network (Figure 5A). This module likely represents the commonly down-regulated genes in HD, rather that changes in tissue composition due to a loss of neurons (see Results section). GO enrichment analysis showed that processes like mitochondrial function, the proteasome, stress response, etc. are probably affected (Table 3). There is extensive literature in PD about mitochondrial dysfunction and the involvement of parkin and PINK1 in quality control and maintenance of mitochondria [60]. Interestingly, *PINK1* is a hub gene in the frontal cortex BA4 module that was enriched for genes involved in mitochondrial function (FC4neg5, Figure 1F and Table 2). As already mentioned in the results section, several genes altered in Alzheimer’s disease are hub genes in the HD/PD consensus network (Figure 6). Collectively these data suggest that some key proteins could underpin the functional deficits observed in various disorders.

The in depth analysis of the hub genes in the different networks, in particular the common ones in all three brain regions uncovered previously identified therapeutic targets (Figure 5). In model systems of HD, for example subcutaneous administration of FGF2 increased neurogenesis [61] and overexpression of metallothioneins conferred neuroprotection against polyglutamine induced excitotoxicity [62]. Furthermore, predicted upstream regulators of the identified network modules e.g. HSF1 [63], NFAT [64], XBP1 [65], ELK1 [66], JUN [67], REST [68], or CREB1 [69] were all shown to modulate neurotoxicity in HD. This clearly shows the power of weighted correlation network analysis for the prediction of therapeutic targets. Therefore, modulation of transcription factors, not yet implicated in HD, like certain members of the STAT transcription factor family (immune response), TCF3 (immune response), TCF12 (lineage-specific gene expression, initiation of neuronal differentiation), EGR1 (differentiation, mitogenesis), EGR2/4 (immune response), IRF1 (immune response, apoptosis), GABPB1 (mitochondrial function), or PAX4 (development, tumorigenesis) could lead to new strategies towards slowing down pathogenesis in Huntington’s disease.

## Conclusions

Using weighted gene correlation network analysis we demonstrate that the Huntington’s disease mutation causes a common signature of gene expression changes in patient brain tissue. We have identified as yet unknown extensive transcriptional dysregulation in the cerebellum of HD patients, similar to that which we have observed in the frontal cortex and caudate nucleus. Additionally, we found that yet unassociated pathways, e.g. global mRNA processing, were dysregulated in HD. Meta analyses of the HD networks and other disorders showed similarities for these disorders with HD, in particular with a high enrichment for inflammatory pathway genes. Lastly, we compared well studied HD mouse models to the human gene expression dataset, which implied that whilst the mouse models mimic some aspects of the disease very well, certain aspects, for example induction of the inflammatory response, were only poorly reflected. Taken together, these approaches allowed us to gain novel insights into the molecular pathogenesis of HD and to pinpoint potential future therapeutic targets.

## Methods

### Microarray datasets and data pre-processing

All datasets were obtained from the EMBL-EBI ArrayExpress [70], or NCBI Gene Expression Omnibus (GEO) [71] websites. Accession numbers for the datasets and experimental details can be found in Table 5. Microarray raw intensity files were MAS5 normalized (Affymetrix Expression Console, Affymetrix, CA, USA) [72] and log2 transformed to obtain the raw data datasets. We used only the HG-U133A data for the main HD dataset [27]. For the neurodegenerative diseases dataset [35], the array files were normalized using the Rosetta error model (Rosetta Biosoftware, WA, USA) and log2 transformed to obtain the raw data dataset. All raw datasets were collapsed to a one probe per gene level using the R function collapseRows [73]. Microarray probes were matched to gene names and Entrez gene IDs (NCBI) of *homo sapiens* genome build hg19 (Consensus CDS, NCBI), if the annotation was not provided by the affymetrix array annotation file (Affymetrix Expression Console, Affymetrix, CA, USA). Probes with ambiguous gene annotations were removed. Outlier samples were removed by a completely unbiased method, which ignores phenotypic traits. To this end the Euclidian distance between samples in a network and their connectivity was calculated. Subsequently, samples with a standardized connectivity of less than −2.5 were removed.

### Weighted gene co-expression network analysis (WGCNA)

All networks were independently constructed from the log2 transformed, pre-processed datasets. In principle, the workflow of the original publications was used [29]. Briefly, the pair wise weighted Pearson correlations between all pairs of genes across all samples were calculated. A signed adjacency matrix was calculated by raising the co-expression matrix to a soft-threshold power to reach approximate scale free topology of the network (R^2^ > 0.9). The powers used were: 15 for cerebellum, 9 for caudate nucleus, 13 for frontal cortex (BA4), 17 for frontal cortex (BA9) and 13 for frontal cortex (BA4 and BA9 combined). A signed topology overlay matrix was calculated based on the transformed connection strengths, which gives a biologically meaningful measurement of the similarity of the co-expression of two genes with all other genes in the network. Highly similarly expressed genes were grouped by applying average linkage hierarchical clustering on the topology overlay matrix. Modules were subsequently identified by the dynamic hybrid tree cut algorithm [74]. Module eigengenes can be seen as representing the first principal component of a module. Modules with highly correlated module eigengenes were merged (*r* >0.8). To identify biological meaningful modules, we correlated the module eigengenes to the HD stage assignment of the samples [27]. Raw *P*-values were adjusted for multiple comparisons with the Benjamini and Hochberg correction using the Bioconductor package multtest [75].

### Consensus network construction

Consensus networks were essentially constructed with the same methodology as described above for weighted gene co-expression network analysis. Briefly, outlier samples were removed from the collapsed raw datasets. Networks were constructed only from genes that were detected by both array types, if applicable. Powers for transformation of the co-expression matrices were (see Table 5 for abbreviations): 10 for AD, 19 for ALS, 28 for DM1, 8 for DM2, 23 for GG, 12 for MS, 16 for PD, 15 for RCC, 9 for SCHIZ, 32 for YAC128, 20 for R6/2 and 20 for *Hdh*Q150. Modules with highly correlated module eigengenes were merged (*r* >0.6). Module eigengenes were subsequently matched to external traits as described before and corrected for multiple hypotheses testing.

### Module preservation statistics

The WGCNA package includes statistical tests to analyze module preservation across different datasets [76]. Preservation is the similarity of interconnections between genes in a module, but also connectivity patterns of individual modules for the two data sets, i.e. high preservation is evidence for densely connected, distinct, and reproducible modules. We calculated 200 permutations of the preservation statistics and generated a Z-summary value by averaging them. The Z-summary indicates if a module is strongly preserved (Z-summary score >10), moderately preserved (Z-summary score 2 < × <10), or not preserved (Z-summary score <2).

### Identification and visualization of hub genes

We used the eigengene based connectivity kME as a measure of module membership. Genes with a high module membership measure are referred to as intramodular hub genes. These genes are representative for the entire module and most likely are biological key players in the respective module. To visualize module structures, we extracted the 50 genes with the highest module membership (nodes) and the strongest 500 gene-gene connections (edges) amongst these from the signed topology overlay matrix. We used Cytoscape [77] to visualize the networks with the strength of the gene-gene correlation as a co-factor.

### Enrichment of upstream regulators and pathway analysis

To analyze enrichment of upstream regulators, like e.g. transcription factors or micro RNAs, we used GO-Elite [78], or WebGestalt [79]. For both, we used the gene lists for the identified network modules as input and all genes in a network as the denominator for the analysis with GO-Elite. The EnsMart65Plus database for *homo sapiens* was used in the GO-Elite analyses.

### Gene ontology analysis

Gene ontology analysis was carried out with the Database for Annotation, Visualization and Integrated Discovery (DAVID) Bioinformatics Resource [80]. A list of all genes in the network analysis was used as a custom background for the gene ontology enrichment analysis. We summarized all gene ontology terms (GO-term) of similar sub-terms into an overarching term. Fold enrichment and Benjamini-Hochberg corrected *P*-values are shown for the respective GO-term cluster.

### Availability of data files

The raw datafiles used in this study [24,27,28,35,81–85] were obtained from the EMBL-EBI ArrayExpress [70], or NCBI Gene Expression Omnibus (GEO) [71] websites. All WGCNA network files, module associations, preservation statistics and consensus data files generated in this publication are available upon request (andreas.neueder@kcl.ac.uk or andreas.neueder@web.de).

## Additional files

Additional file 1:
**Illustrates the correlation between gene significance and eigengene based connectivity (kME).**
Additional file 2:
**Figure illustrating the remaining cerebellum network modules.**
Additional file 3:
**Figure illustrating the remaining frontal cortex BA4 network modules.**
Additional file 4:
**Figure illustrating the remaining caudate nucleus network modules.**
Additional file 5:
**Figure illustrating the remaining module of the consensus network analysis of HD and PD.**
Additional file 6:
**Figure illustrating the consensus network analysis of HD and GG.**
Additional file 7:
**Figure illustrating the consensus network analysis of HD and RCC.**
Additional file 8:
**Figure illustrating the consensus network analysis of HD and DM1.**
Additional file 9:
**Figure illustrating the consensus network analysis of HD and DM2.**
Additional file 10:
**Figure illustrating the WGCNA analysis of the HD frontal cortex dataset with BA4 and BA9 regions combined.**
Additional file 11:
**Table describing the gene ontology enrichment and upstream regulator analysis of the frontal cortex network with BA4 and BA9 regions combined.**

## Abbreviations

AD: Alzheimer’s disease
ALS: Amyotrophic lateral sclerosis
BA4/9: Brodmann area 4/9
CB: Cerebellum
CN: Caudate nucleus
DCM: Dilated cardiomyopathy
DM1: Myotonic dystrophy type 1
DM2: Myotonic dystrophy type 2
DMD: Duchenne Muscular Dystrophy
FC: Frontal cortex
GG: Ganglioglioma
GO: Gene ontology
HD: Huntington’s disease
HTT: Huntingtin
MS: Multiple sclerosis
PD: Parkinson’s disease
SCHIZ: Schizophrenia
WGCNA: Weighted gene correlation network analysis
RCC: Renal cell carcinoma
