# Supplementary material for: A common gene expression signature in Huntington’s disease patient brain regions
Source: BMC Med Genomics. 2014 Oct 30;7:60. doi: 10.1186/s12920-014-0060-2 (PMC4219025; doi:10.1186/s12920-014-0060-2)
Supplement: Additional file 1: — Illustrates the correlation between gene significance and eigengene based connectivity (kME). [file 12920_2014_60_MOESM1_ESM.pdf]

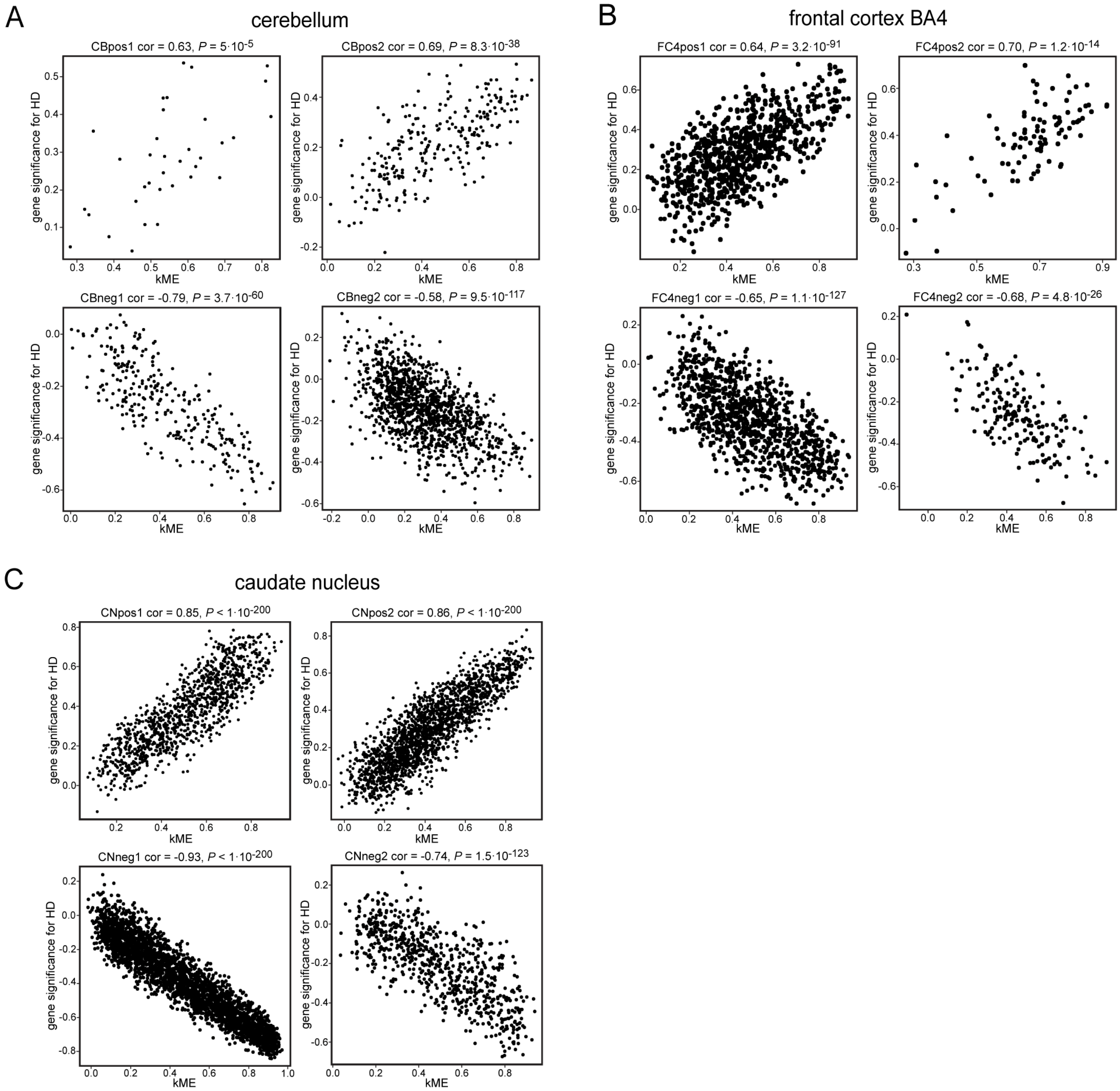

Additional data file 1. Correlations of eigengene based connectivity (kME) versus the gene significance for HD in the three brain tissue networks. The four modules with the highest absolute correlation with HD are shown for each network. The highly significant correlations indicate the occurrence of hub genes, i.e. genes with high absolute values for gene significance for HD and kME. cor = correlation.
