# Supplementary material for: A common gene expression signature in Huntington’s disease patient brain regions
Source: BMC Med Genomics. 2014 Oct 30;7:60. doi: 10.1186/s12920-014-0060-2 (PMC4219025; doi:10.1186/s12920-014-0060-2)
Supplement: Additional file 4: — Figure illustrating the remaining caudate nucleus network modules. [file 12920_2014_60_MOESM4_ESM.pdf]

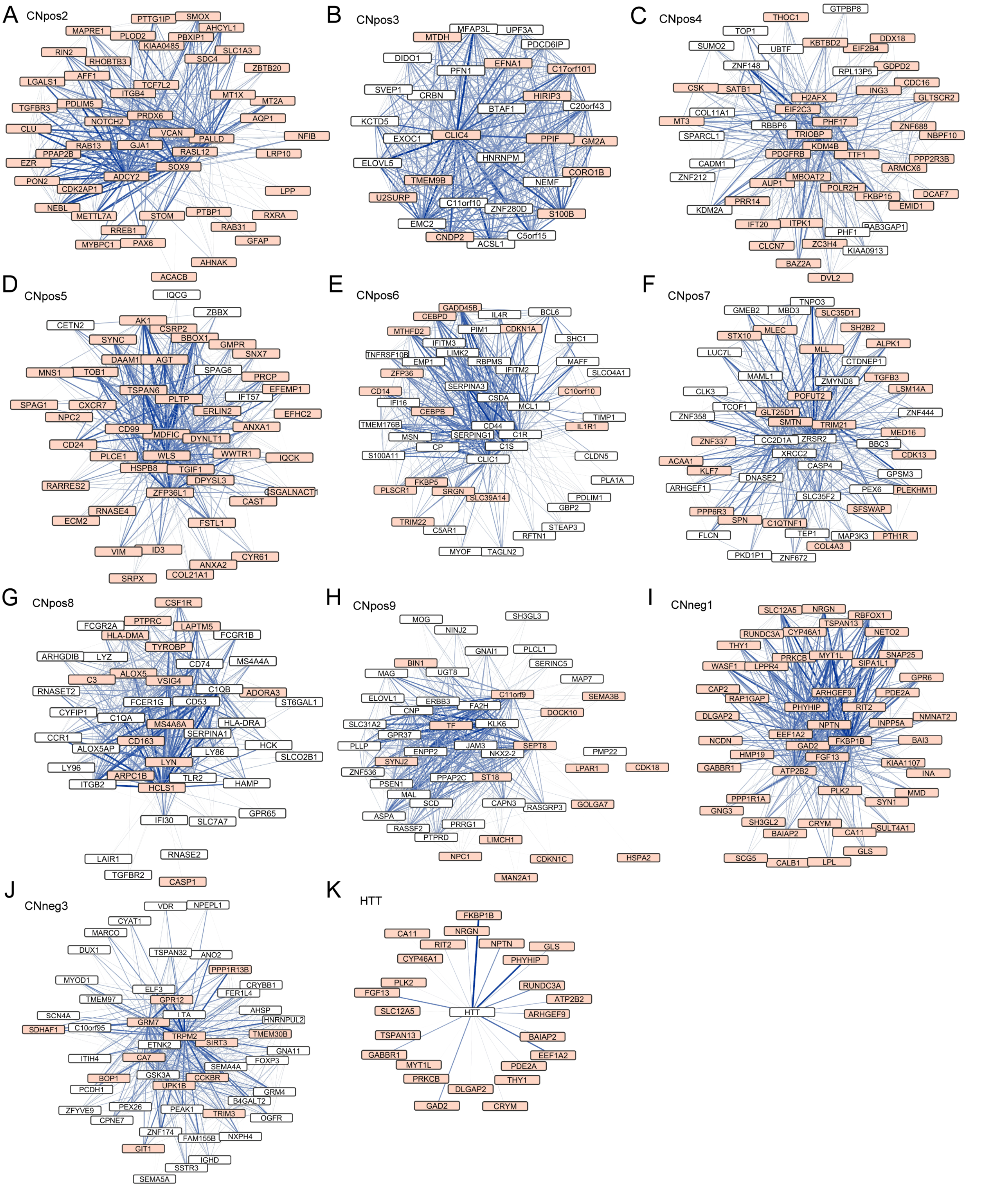

Additional data file 4. Visualization of hub genes in caudate nucleus network modules. (**A** - **K**) The 50 most connected genes (nodes) and the 500 strongest gene-gene interactions (edges) in each module are shown. The width and the color saturation of the lines (edges) correspond to the weight of the interactions. The orange highlighted nodes correspond to genes that were also statistically significantly dysregulated [27]. The hub genes for the positively correlated modules **A** to **H** and for the negatively correlated modules **I** to **J** are shown. HTT is a member of the CNneg1 module in the caudate nucleus network. Its 25 strongest interactions are shown in **K**.
