# Supplementary material for: A common gene expression signature in Huntington’s disease patient brain regions
Source: BMC Med Genomics. 2014 Oct 30;7:60. doi: 10.1186/s12920-014-0060-2 (PMC4219025; doi:10.1186/s12920-014-0060-2)
Supplement: Additional file 6: — Figure illustrating the consensus network analysis of HD and GG. [file 12920_2014_60_MOESM6_ESM.pdf]

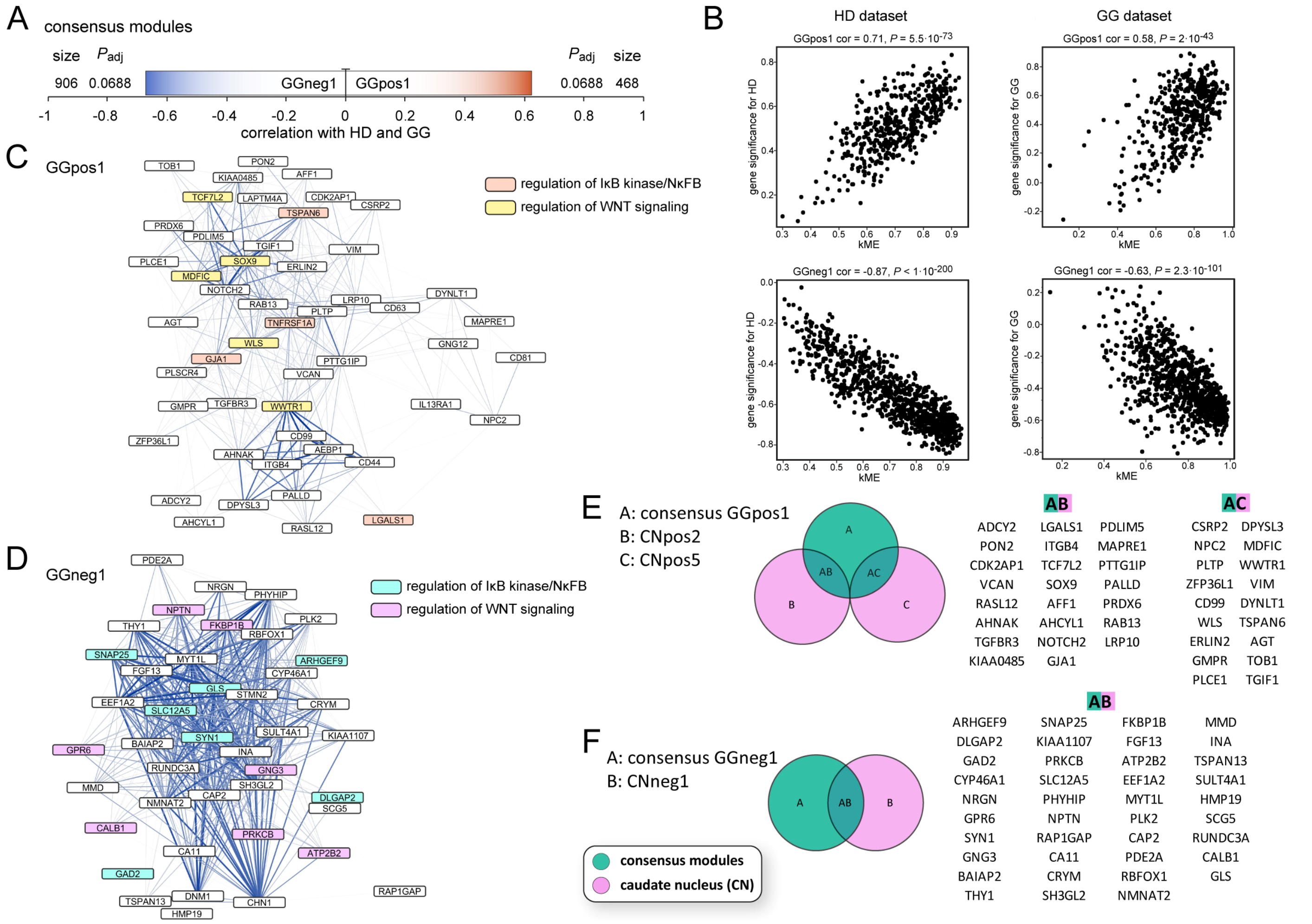

Additional data file 6. WGCNA analysis of the HD/GG consensus dataset. (**A**) Visualization of modules that are highly correlated with Huntington's (HD) and ganglioglioma (GG) disease state. Size is the number of genes for each module. P_{adj} gives the Benjamini Hochberg corrected significance value of correlation with HD/GG for each module. (**B**) Correlations of eigengene based connectivity (kME) versus the gene significance for HD and GG. cor = correlation. (**C** and **D**) Visualization of hub genes in HD/GG consensus network modules. The 50 most connected genes (nodes) and the 500 strongest gene-gene interactions (edges) in each module are shown. The width and the color saturation of the lines (edges) correspond to the weight of the interactions. (**E** and **F**) Hub gene comparison of HD/GG consensus modules versus modules of the HD caudate nucleus (CN) dataset. Venn diagrams show the overlap of hub genes in the respective consensus modules with HD caudate nucleus modules.
