# Supplementary material for: A common gene expression signature in Huntington’s disease patient brain regions
Source: BMC Med Genomics. 2014 Oct 30;7:60. doi: 10.1186/s12920-014-0060-2 (PMC4219025; doi:10.1186/s12920-014-0060-2)
Supplement: Additional file 9: — Figure illustrating the consensus network analysis of HD and DM2. [file 12920_2014_60_MOESM9_ESM.pdf]

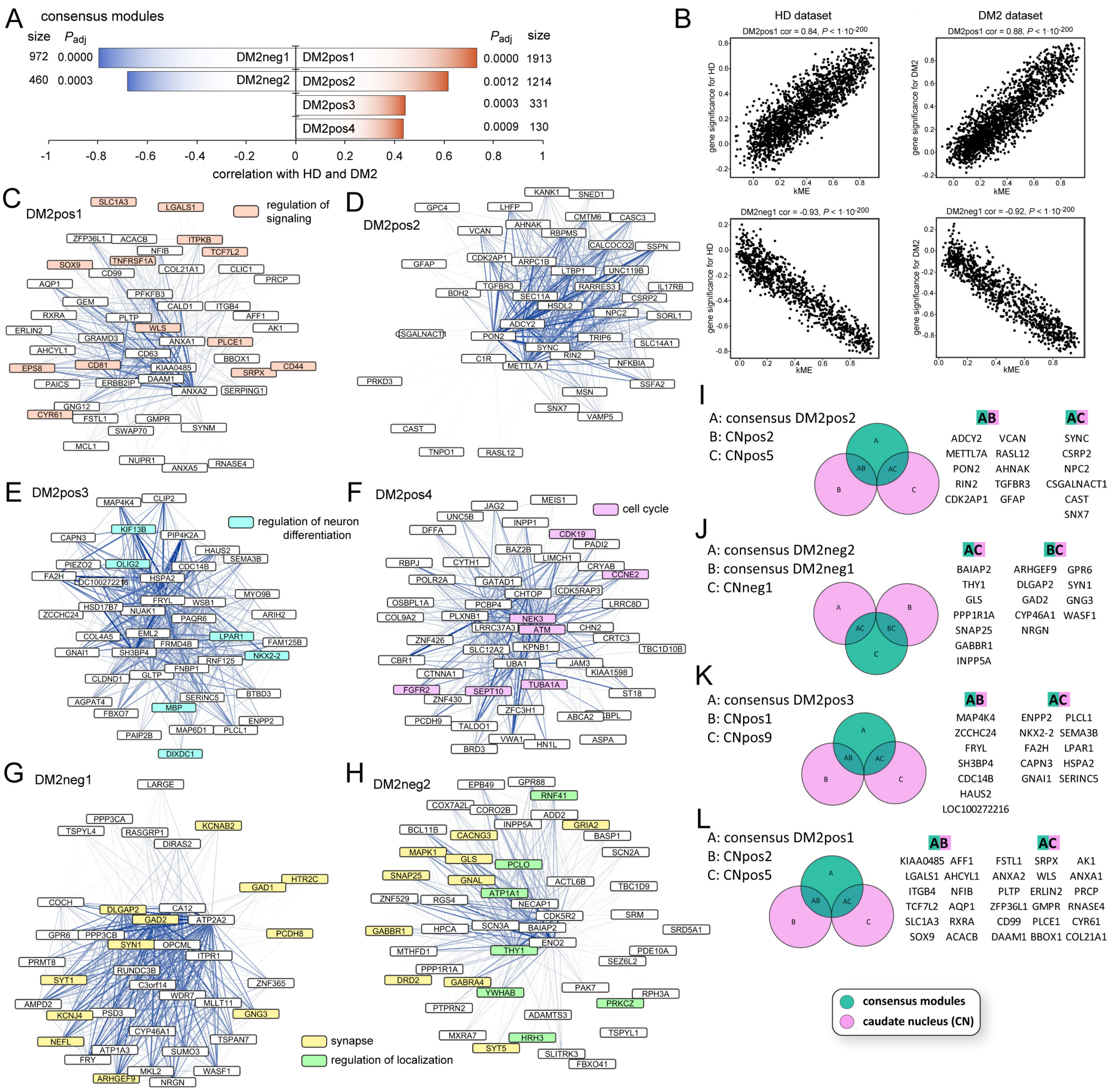

Additional data file 9. WGCNA analysis of the HD/DM2 consensus dataset. (**A**) Visualization of modules that are highly correlated with Huntington's (HD) and myotonic dystrophy type 2 (DM2) disease state. Size is the number of genes for each module. P_{adj} gives the Benjamini Hochberg corrected significance value of correlation with HD/DM2 for each module. (**B**) Correlations of eigengene based connectivity (kME) versus the gene significance for HD and DM2. The two modules with the highest absolute correlation are shown for each disease dataset. cor = correlation. (**C** and **D**) Visualization of hub genes in HD/DM2 consensus network modules. The 50 most connected genes (nodes) and the 500 strongest gene-gene interactions (edges) in each module are shown. The width and the color saturation of the lines (edges) correspond to the weight of the interactions. (**E** - **H**) Hub gene comparison of HD/DM2 consensus modules versus modules of the HD caudate nucleus (CN) dataset. Venn diagrams show the overlap of hub genes in the respective consensus modules with HD caudate nucleus modules. Only consensus modules with an overlap of 5 or more genes to CN modules are shown.
