# Supplementary material for: A common gene expression signature in Huntington’s disease patient brain regions
Source: BMC Med Genomics. 2014 Oct 30;7:60. doi: 10.1186/s12920-014-0060-2 (PMC4219025; doi:10.1186/s12920-014-0060-2)
Supplement: Additional file 10: — Figure illustrating the WGCNA analysis of the HD frontal cortex dataset with BA4 and BA9 regions combined. [file 12920_2014_60_MOESM10_ESM.pdf]

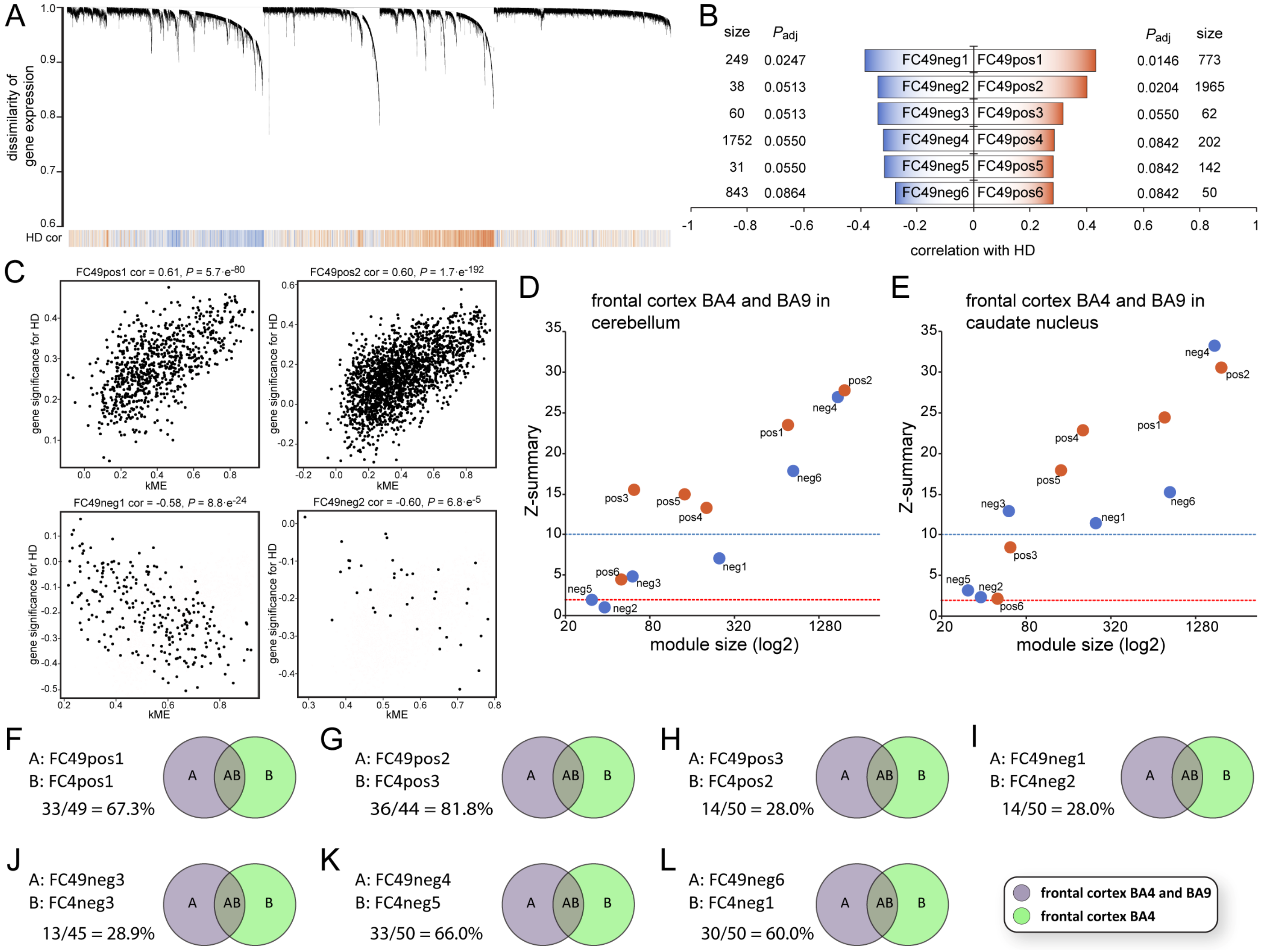

Additional data file 10. WGCNA analysis of the HD frontal cortex dataset with BA4 and BA9 regions combined. (**A**) Hierarchical cluster tree of the average linkage in the dissimilarity topological overlap matrix. Each vertical line correlates to a gene. The height is a measure for the dissimilarity based on the topological overlap. The band under the dendrogram indicates the correlation with HD (HD cor) based on the gene significance for each gene. Red is positively correlated with HD stage, blue is negatively correlated. (**B**) Visualization of modules that are highly correlated with HD. Size is the number of genes for each module. P_{adj} gives the Benjamini Hochberg corrected significance value of correlation with HD for each module. (**C**) Correlations of eigengene based connectivity (kME) versus the gene significance for HD. The four modules with the highest absolute correlation with HD are shown. cor = correlation. (**D and E**) Preservation analysis. The Z-summary is a measure for module preservation. Values less than 2 (red lines) indicate no preservation, between 2 and 10 (blue lines) module structures are preserved and above 10 the module structure is highly preserved. The gene number for each module is given in brackets after the module color. (**D**) Preservation analysis of frontal cortex BA4 and BA9 regions modules in the cerebellum dataset. (**E**) Preservation analysis of frontal cortex BA4 and BA9 regions modules in the caudate nucleus dataset. (**F** - **L**). Hub gene comparison of the HD frontal cortex dataset BA4 region modules versus modules of the frontal cortex dataset with BA4 and BA9 regions combined. Venn diagrams show the percent overlap of hub genes in the respective modules (number of genes in the intersection / number of genes in the frontal cortex dataset BA4 region network modules).
