# Supplementary material for: A common gene expression signature in Huntington’s disease patient brain regions
Source: BMC Med Genomics. 2014 Oct 30;7:60. doi: 10.1186/s12920-014-0060-2 (PMC4219025; doi:10.1186/s12920-014-0060-2)
Supplement: Additional file 11: — Table describing the gene ontology enrichment and upstream regulator analysis of the frontal cortex network with BA4 and BA9 regions combined. [file 12920_2014_60_MOESM11_ESM.pdf]

Additional data file 11.

Gene ontology enrichment for the frontal cortex combined BA4 and BA9 regions network.

module	cor.	GO-term (DAVID)	potential regulators
BA49pos1	up	vasculature development (6.01, 0.000)<br>immune response (4.51, 0.000)	p53 (0.036)[1], NFκB (0.036)[1], miR124 (0.036)[1], SRF (0.036)[1], ETS2 (0.008)[2], JUN (0.012)[2], IRF8 (0.012)[2], STAT3 (0.024)[2]
BA49pos2	up	lipid metabolism (4.17, 0.006)<br>negative regulation of gene expression (3.94, 0.011)<br>amino acid catabolic process (3.65, 0.018)<br>chromatin organization (3.17, 0.019)	
BA49pos3	up	chromatin modification (1.28, 0.08)	AR (0.003)[2], FOXN1 (0.003)[2], E2F (0.010)[2], NFATC (0.034)[2], HSF1 (0.034)[2], PTF1A (0.034)[2], MEIS1 (0.044)[2], MEF2 (0.044)[2]
BA49pos4	up	regulation of transcription (2.35, 0.008)	**miR124** (0.027)[1], ETS1 (0.027)[1], **miR124A** (0.000)[2]
BA49pos5	up	cytoplasmic ribosome (55.74, 0.000)	ELK1 (0.001)[2], NRF2 (0.005)[2]
BA49pos6	up	regulation of transcription (1.7, 0.028)	
BA49neg1	down	synapse (2.24, 0.140)	ESRRA (0.000)[2], SF1 (0.000)[2], NFE2 (0.005)[2], REST (0.012)[2], JUN (0.032)[2]
BA49neg2	down	potassium transport (0.98, 0.93)	
BA49neg3	down	calmodulin binding (1.49, 0.6)	GTF3A (0.010)[2], PAX4 (0.011)[2], PPARA (0.011)[2], SREBF1 (0.011)[2], MYOG (0.011)[2], STAT3 (0.041)[2], TCF3 (0.041)[2], HNF4A (0.048)[2]
BA49neg4	down	mitochondrion (8.67, 0.000)<br>proteasome/protein catabolic process (5.29, 0.000)<br>protein transport (4.04, 0.000)<br>signalosome (3.43, 0.000)<br>chaperones (2.76, 0.013)<br>mitochondrial ribosome (2.5, 0.005)	**NRF1** (0.004)[1], **CREB** (0.004)[1], YY1 (0.004)[1]<br>ELK1 (0.000)[2], SP1 (0.000)[2], E4F1 (0.000)[2], GABPA (0.002)[2], SF1 (0.003)[2], TCF11 (0.008)[2], NFY (0.012)[2], **NRF1** (0.012)[2], ATF3 (0.019)[2], NRF2 (0.019)[2], NFE2 (0.028)[2], **CREB** (0.029)[2], JUN (0.029)[2]
BA49neg5	down	neuron development (0.65, 1.0)	
BA49neg6	down	protein catabolic process (7.77, 0.000)<br>protein transport (3.17, 0.02)	
HTT	n.a.	cytoskeletal protein binding (1.57, 0.43)	

Gene ontology (GO) enrichment for the frontal cortex BA4 and BA9 region network. Genes in the identified modules were analyzed using DAVID. The sign of the correlation (cor) with HD and the over-represented GO-terms are shown. The first number in brackets after the GO-term is the respective fold enrichment, the second number the adjusted *P*-value, as determined by DAVID. All significantly enriched (adjusted $P < 0.05$) GO-terms are shown. In cases where no significantly enriched GO-term was identified, the GO-term with the highest fold enrichment is shown. Potential regulators of a module were identified using [1]GO-Elite, or [2]WebGestalt. Adjusted *P*-values are given in brackets after the name. Regulators that were identified by both tools are highlighted in bold. HTT is part of a module, which is not correlated with HD in the frontal cortex BA4 and BA9 region network. The GO-term enrichment for 100 genes with the highest correlation with HTT is shown.
