# Supplementary material for: A common gene expression signature in Huntington’s disease patient brain regions
Source: BMC Med Genomics. 2014 Oct 30;7:60. doi: 10.1186/s12920-014-0060-2 (PMC4219025; doi:10.1186/s12920-014-0060-2)
Supplement: Additional file 5: — Figure illustrating the remaining module of the consensus network analysis of HD and PD. [file 12920_2014_60_MOESM5_ESM.pdf]

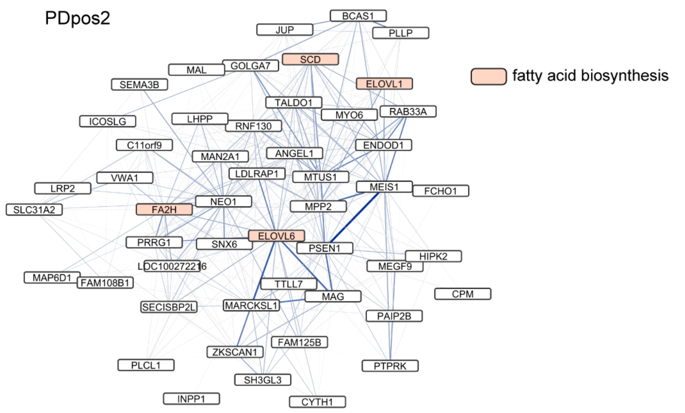

Additional data file 5. Visualization of hub genes in the PDpos2 module of the HD/PD consensus network. The 50 most connected genes (nodes) and the 500 strongest gene-gene interactions (edges) are shown. The width and the color saturation of the lines (edges) correspond to the weight of the interactions.
